# IGF1R Contributes to Cell Proliferation in *ALK*-Mutated Neuroblastoma with Preference for Activating the PI3K-AKT Signaling Pathway

**DOI:** 10.3390/cancers15174252

**Published:** 2023-08-25

**Authors:** Jikui Guan, Marcus Borenäs, Junfeng Xiong, Wei-Yun Lai, Ruth H. Palmer, Bengt Hallberg

**Affiliations:** 1 Institute of Pediatric Medicine, Children’s Hospital Affiliated to Zhengzhou University, Zhengzhou 450018, China; 2 Department of Medical Biochemistry and Cell Biology, Institute of Biomedicine, Sahlgrenska Academy, University of Gothenburg, SE-40530 Gothenburg, Swedenruth.palmer@gu.se (R.H.P.); bengt.hallberg@gu.se (B.H.); 3 Department of Biochemistry and Molecular Biology, Shanxi Medical University, Taiyuan 030001, China

**Keywords:** anaplastic lymphoma kinase, adaptor protein, lorlatinib, GSK1904529A, linsitinib

## Abstract

**Simple Summary:**

The insulin-like growth factor 1 receptor (IGF1R) is a receptor tyrosine kinase (RTK) widely expressed in many cancers, including the pediatric cancer neuroblastoma. Aberrant activation of the anaplastic lymphoma kinase (ALK) RTK by activating point mutations or amplification is identified in 5–12% of neuroblastomas. Here, we investigated IGF1R in ALK-driven neuroblastoma, with the aim of understanding its contribution and exploring its potential for targeted therapy. Using ALK-driven neuroblastoma cell lines, we show that *ALK*-mutated cells are more sensitive to IGF1R inhibition than *ALK*-amplified cells, and a synergistic effect is obtained when combining ALK and IGF1R inhibitors. Mechanistically, in *ALK*-mutated neuroblastoma cells, both ALK and IGF1R contribute significantly to the activation of the downstream PI3K-AKT and RAS-MAPK signaling pathways. Our results suggest that differential activation of signaling downstream of the ALK and IGF1R pathways is in part due to preferential recruitment of adaptor proteins.

**Abstract:**

Aberrant activation of anaplastic lymphoma kinase (ALK) by activating point mutation or amplification drives 5–12% of neuroblastoma (NB). Previous work has identified the involvement of the insulin-like growth factor 1 receptor (IGF1R) receptor tyrosine kinase (RTK) in a wide range of cancers. We show here that many NB cell lines exhibit IGF1R activity, and that IGF1R inhibition led to decreased cell proliferation to varying degrees in ALK-driven NB cells. Furthermore, combined inhibition of ALK and IGF1R resulted in synergistic anti-proliferation effects, in particular in *ALK*-mutated NB cells. Mechanistically, both ALK and IGF1R contribute significantly to the activation of downstream PI3K-AKT and RAS-MAPK signaling pathways in *ALK*-mutated NB cells. However, these two RTKs employ a differential repertoire of adaptor proteins to mediate downstream signaling effects. We show here that ALK signaling led to activation of the RAS-MAPK pathway by preferentially phosphorylating the adaptor proteins GAB1, GAB2, and FRS2, while IGF1R signaling preferentially phosphorylated IRS2, promoting activation of the PI3K-AKT pathway. Together, these findings reveal a potentially important role of the IGF1R RTK in *ALK*-mutated NB and that co-targeting of ALK and IGF1R may be advantageous in clinical treatment of *ALK*-mutated NB patients.

## 1. Introduction

Insulin-like growth factor 1 receptor (IGF1R) is a receptor tyrosine kinase (RTK) expressed in many cells and tissues [1]. Structurally, it is closely related to the insulin receptor (InsR) RTK and is a member of the InsR superfamily [2]. Binding of the IGF-1 ligand leads to activation of IGF1R and the downstream phosphatidylinositol 3-kinase (PI3K)-AKT and RAS-mitogen-activated protein kinase (MAPK) pathways, through which IGF1R regulates a variety of biological processes like cell proliferation, cell differentiation, and cell survival [1]. Dysregulation of IGF1R signaling has been reported in many cancers, especially breast cancer. Despite many studies supporting the role of IGF1R in tumorigenesis, clinical trials targeting its activity have not been successful [3,4,5,6], indicating that IGF1R might not be the key oncogenic driver in these cancers.

Neuroblastoma (NB) is a childhood cancer arising from the developing sympathetic nerve system, characterized by a complex genetic background and dramatically heterogeneous clinical outcomes [7]. In 2008, activating mutations in the kinase domain of the anaplastic lymphoma kinase (ALK) RTK were reported as oncogenic drivers in both sporadic and familial NB cases [8,9,10,11,12]. Like IGF1R, ALK also belongs to the InsR superfamily, characterized by the “Y*XXX*YY” motif within their activation loops of the kinase domain [13,14]. Oncogenic *ALK* alterations, including activating mutations, are identified in approximately 5–10% of primary NBs, and *ALK* amplification in 1–2% of cases [15,16,17]. Furthermore, *ALK* mutations were identified in up to 20–25% of relapsed NB cases [18]. The majority of *ALK*-activating mutations occur at three ‘hotspot’ residues within the kinase domain (F1174, F1245, and R1275) [17,19,20]. These oncogenic alterations lead to constitutive ALK activity and activation of downstream signaling pathways, including the PI3K-AKT, RAS-MAPK, JAK-STAT, CRKL-C3G-RAP1, and PLCγ-DAG-PKC pathways, resulting in enhanced cell proliferation, survival, angiogenesis, and migration [21,22,23].

The high incidence of *ALK* alterations and the presence of tyrosine kinase domain make ALK an important therapeutic target for ALK-driven NBs. Several ALK tyrosine kinase inhibitors (TKIs) are in clinical use. The first generation ALK inhibitor, crizotinib, is less effective in treating ALK-driven NBs due to mutations in *ALK* itself that can hinder efficient binding as compared with the wild-type ALK kinase domain [24,25]. The second and the third generations of ALK TKIs, such as ceritinib, alectinib, brigatinib, and lorlatinib, have been widely tested in both preclinical and clinical NB settings and have shown greater potency than crizotinib [26,27,28,29,30,31,32,33,34]. Although now well understood, a pattern of resistance to ALK TKI treatment in NB is emerging, and the underlying mechanisms include: mutation of *ALK* itself [35], reactivation of the RAS-MAPK pathway [36], epigenetic reprogramming [37], and gene amplification of cyclin-dependent kinases (CDKs) and other RTKs [38]. ALK, the neurotrophin receptor RET, and the TRK RTKs have been reported to be involved in both neural crest development and NB tumorigenesis [39,40,41,42,43,44,45,46]. RET has been shown to be regulated by ALK through the ERK-ETV5-RET pathway to drive oncogenesis in ALK-driven NB [46]. ALK also regulates RET through transactivation, and loss of RET promotes mesenchymal identity in NB cells [44]. We and others have also identified activation of IGF1R signaling in NB cells employing phosphoproteomic analyses [47,48]. 

Since multiple RTKs are expressed in NB, it is reasonable to think that some of them may be able to compensate for the impaired growth signaling due to inhibition of one RTK (for example, ALK), resulting in poor treatment response or resistance. Therefore, exploration of druggable RTKs is motivated to test whether their inhibition can enhance the efficacy of ALK-targeted therapy. In this paper, we investigated the contribution of IGF1R activity to the proliferation of ALK-driven NB and explored the potential of combined inhibition of ALK and IGF1R for treating this group of NB. We show that IGF1R inhibition leads to decreased cell proliferation to varying extents in ALK-driven NB cells. Furthermore, combined inhibition of ALK and IGF1R results in synergistic anti-proliferation effects, particularly in *ALK*-mutated NB cells. We further identify differential use of downstream adaptor proteins by either ALK or IGF1R in ALK-driven NB cells, leading to differential activation of downstream signaling pathways. Finally, we demonstrate that IGF1R plays an important role in *ALK*-mutated NB and suggest that co-targeting ALK and IGF1R might be a promising treatment option for this group of NB patients.

## 2. Materials and Methods

### 2.1. Antibodies, Inhibitors, and Reagents

The primary antibodies used were: anti-IGF1Rβ (D23H3), anti-pIGF1Rβ (Y1135/1136) (19H7), anti-ALK (D5F3), anti-pALK (Y1278) (D59G10), anti-pAKT (S473), anti-pERK1/2, anti-pS6 (S240/244), anti-p4E-BP1 (T37/46), anti-PARP (46D11), anti-IRS2, anti-PI3 kinase p85 (19H8), anti-MYCN (D4B2Y), anti-pGAB1 (Y627) (C32H2), anti-pGAB2 (Y452), and anti-pFRS2 (Y436), all from Cell Signaling Technology (Danvers, MA, USA); and anti-β-Tubulin (BT7R, 1:5000) and anti-pIRS1 Y612/pIRS2 Y653 from Invitrogen Antibodies (ThermoFisher Scientific, Waltham, MA, USA). The anti-IRS2 (B-5) and anti-PI3 kinase p85 (U5) antibodies used for immunoprecipitation were from Santa Cruz Biotechnology (Dalla, TX, USA) and Invitrogen Antibodies, respectively. The secondary antibodies were goat anti-mouse IgG horse radish peroxidase (HRP) conjugate and goat anti-rabbit IgG HRP conjugate purchased from Invitrogen Antibodies. The ALK inhibitor lorlatinib (T3061) and IGF1R inhibitors GSK1904529A (T6003) and linsitinib (T6017) were purchased from TargetMol Chemicals Inc. (Boston, MA, USA). Recombinant ALKAL2 was produced by IBA Lifesciences (Göttingen, Germany). Recombinant human IGF-1 was purchased from Sigma-Aldrich of the Merck Group (Darmstadt, Germany). The Proteome Profiler Human Phospho-RTK Array Kit (ARY001B) was purchased from R&D Systems (Minneapolis, MN, USA).

### 2.2. Cell Lines and Cell Culture

Neuroblastoma cell lines used in this study included CLB-BAR (*ALK*-amplified), CLB-GAR (*ALK* R1275Q), CLB-GE (*ALK* F1174V), NB1 (*ALK*-amplified), SH-SY5Y (*ALK* F1174L, *KRAS* G12V), SK-N-AS (*NRAS* Q61K), and BE2C (*NF1*-low). All CLB cell lines were from Centre Léon Bérard under a material transfer agreement. NB1 was purchased from RIKEN BioResource Research Center (Tsukuba, Ibaraki, Japan). SH-SY5Y (CRL-2266), SK-N-AS (CRL-2137) and BE2C (CRL-2268) were purchased from ATCC (Manassas, VA, USA). Detailed genetic information is provided in Supplementary Figure S1B as well as described previously [49]. Cells were cultured in RPMI 1640 medium supplemented with 10% fetal bovine serum (FBS) and a mixture of 1% penicillin/streptomycin at 37 °C under a humidified atmosphere with 5% CO_2_. 

### 2.3. Cell Growth and Proliferation Assay

Cells were seeded at 1 × 10^4^ per well in 48-well plates and treated with serial dilutions of the indicated inhibitors. To test the sensitivity to IGF1R inhibitor GSK1904529A, the Sartorius Incucyte S3 Live Cell Analysis System (Essen Bioscience, Ann Arbor, MI, USA) was used to monitor cell growth every 6 h over a time course of 96 h. Growth curves were generated by normalizing the cell growth area per image to 0 h.

To test the inhibitory effect on cell proliferation, cell viability was measured by resazurin assay after 72 h treatment. Inhibition curves were generated with GraphPad Prism 9.0 by fitting data to a log10 (inhibitor concentration) vs. normalized response (variable slope) equation, or by fitting data to inhibitor concentration vs. normalized response (variable slope) equation. IC50 values were calculated during generation of the curves. For synergy calculation, a combination index (CI) was used and the CI values were determined using the CompuSyn software 1.0.1 according to the method previously described for combinations of different drug concentrations [50]. All experiments were repeated at least three times. 

### 2.4. Colony Formation Assay

Cells were seeded at 1 × 10^4^ per well in 6-well plates and cultured with medium containing inhibitors or DMSO control. The medium was changed every 3 days to remove dead cells. After 14 days, the medium was removed, and cells were washed with cold 1× PBS and then fixed with cold methanol for 10 min. Cell colonies were stained with 0.1% crystal violet for 20 min at room temperature, washed with distilled water to remove background staining, and then air-dried. 

### 2.5. siRNAs and Cell Transfection

Silencer Select siRNAs, including negative control siRNA and two validated human IGF1R siRNAs (s7211, s7212), were purchased from ThermoFisher Scientific (Waltham, MA, USA). CLB-GAR and CLB-GE cells were transfected with siRNAs using Lipofectamine RNAiMAX transfection reagent (ThermoFisher Scientific, Waltham, MA, USA) according to the manufacturer’s protocols. Cells transfected with negative control siRNA were used as controls. Two days post-transfection, cells were split for colony formation assay and immunoblotting analysis. For colony formation assay, 1 × 10^4^ cells were replated in 6-well plates in duplicate and cultured for another 5 days. Then they were subjected to another round of siRNA transfection and cultured for another 7 days before fixation and staining with crystal violet. The remaining cells were harvested for immunoblotting analysis to examine the knockdown of IGF1R and its effect on downstream signaling.

### 2.6. Cell Lysis, Immunoprecipitation, and Immunoblotting Analysis

NB cell lines cultured under normal conditions were lysed with RIPA buffer (ThermoFisher Scientific, Waltham, MA, USA) containing cOmplete protease inhibitor and PhosSTOP phosphatase inhibitor cocktails (Roche Life Sciences, Basel, Switzerland). Cell extracts were measured with a Pierce BCA Protein Kit (ThermoFisher Scientific, Waltham, MA, USA). For immunoprecipitation, NB cells cultured in 10 cm dishes were stimulated with 1 µg/mL ALKAL2 or 200 ng/mL IGF-1 for 15 min prior to lysis with RIPA buffer as described above. Cells without stimulation were used as control. Clarified cell lysates were incubated with either anti-IRS2 antibody or anti-PI3 Kinase p85 antibody at 4 °C for 4 h and then with Protein G Sepharose 4 Fast Flow (Cytiva, Uppsala, Sweden) at 4 °C overnight. Sepharose beads were then washed with lysis buffer 5 times prior to elution of the IP products in 1× SDS sample buffer. 

Cells treated with inhibitors, siRNAs, or growth factors as indicated in the figures were directly lysed in 1× SDS sample buffer containing cOmplete protease inhibitor and PhosSTOP phosphatase inhibitor cocktails and boiled at 90 °C for 10 min. Whole cell lysates were then clarified by centrifugation at 14,000 rpm for 5 min before being separated by 8% SDS-PAGE gel and blotted with antibodies as indicated in the figures.

Precleared lysates were separated on SDS-PAGE, transferred to polyvinylidene difluoride membranes (Millipore, Burlington, VT, USA), blocked with 3% bovine serum albumin (BSA) solution, and incubated with primary antibodies diluted in 3% BSA solution overnight at 4 °C. Blots were then incubated with secondary antibodies at room temperature with shaking for 1 h. SuperSignal West Pico PLUS chemiluminescent substrates (ThermoFisher Scientific, Waltham, MA, USA) were used for detection, and membranes were scanned using the LI-COR Odyssey Fc Imaging System (LI-COR Biosciences, Lincoln, NE, USA).

### 2.7. Cell Cycle Analysis

Cell cycle analysis was performed with NucleoCounter NC-3000 image cytometer (ChemoMetec A/S, Allerod, Denmark). In brief, cells were seeded at 1 × 10^5^ per well in 6-well plates and treated with single inhibitors or combinations as indicated in the figures for 48 h. Cells were then harvested and analyzed following the manufacturer’s protocol. The percentage of each cell population was measured with the accompanying NucleoView software NC-3000 1.3.19.0. Results represent mean ± S.D. from at least three independent experiments.

## 3. Results

### 3.1. IGF1R Activity Is Present in ALK-Driven NB Cells

Quantitative phosphoproteomic analysis has shown that both IGF1R and RET RTKs are activated in NB10, a *MYCN*-amplified NB cell line [47]. We also found that treatment with ALK inhibitors leads to reduced phosphorylation of IGF1R and other RTKs in ALK-driven NB cell lines [48]. More recently, we have shown that the RET RTK can also be phosphorylated in response to ALK activation [44]. 

**Figure 1:**
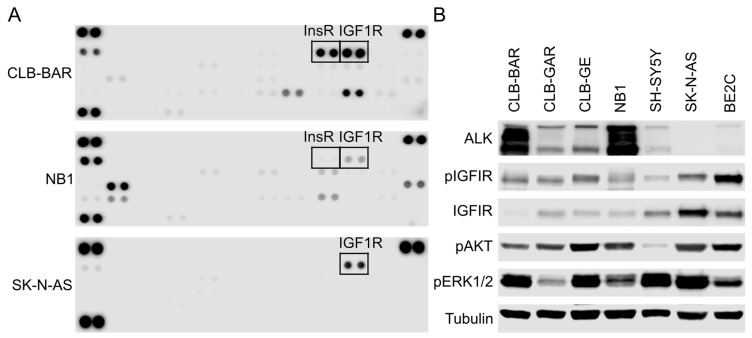
IGF1R is active in ALK-driven NB cell lines. (**A**) A human Phospho-RTK array was used to detect active RTKs in CLB-BAR, NB1, and SK-N-AS cell lysates. Boxed dots indicate pIGF1R and pInsR. (**B**) Immunoblotting analysis of ALK, IGF1R, pIGF1R, as well as downstream pAKT and pERK1/2 signaling in ALK-driven NB cell lines, including CLB-BAR (*ALK*-amplified), CLB-GAR (*ALK* R1275Q), CLB-GE (*ALK* F1174V), NB1 (*ALK*-amplified), and SH-SY5Y (*ALK* F1174L), as well as the non-ALK-driven cell lines SK-N-AS (*NRAS* Q61K) and BE2C (*NF1*-low). Tubulin was used as loading control. The uncropped blots are shown in File S1.

To further identify RTKs active in ALK-driven NB cells, we employed a human phospho-RTK array (layout in Supplementary Figure S1A), observing that both InsR and IGF1R were active in CLB-BAR and NB1 cells, and the signal intensity of pInsR was weaker than that of pIGF1R, particularly in NB1 cells (Figure 1A). Phosphorylation of IGF1R but not InsR was also detectable in SK-N-AS (Figure 1A), a *NRAS*^Q61K^-driven NB cell line that expresses wild-type ALK. Interestingly, we observed more active RTKs in the two ALK-driven cell lines compared with non-ALK-driven SK-N-AS (Figure 1A), which may reflect complex transactivation regulation among these RTKs, consistent with our previous findings [44,48]. 

To verify the phospho-RTK array results, immunoblotting was performed on five ALK-driven NB cell lines: CLB-BAR (*ALK*-amplified), CLB-GAR (*ALK* R1275Q), CLB-GE (*ALK* F1174V), NB1 (*ALK*-amplified), and SH-SY5Y (*ALK* F1174L), as well as two non-ALK-driven NB cell lines: SK-N-AS and BE2C (detailed genetic backgrounds in Supplementary Figure S1B). In agreement with our previous findings, we could confirm that IGF1R was expressed and active in all seven cell lines (Figure 1B). A previous study employing the same phospho-RTK array also demonstrated the presence of IGF1R activity in SH-SY5Y as well as another two non-ALK-driven NB cell lines SK-N-BE(2) and IMR-32 [51]. CLB-BAR and NB1, two *ALK*-amplified cell lines, exhibit higher ALK expression levels than CLB-GAR, CLB-GE, and SH-SY5Y, where *ALK* is mutated but not heavily amplified. All seven cell lines showed hyperactivation of AKT and ERK1/2, suggesting active RTK signaling to downstream PI3K-AKT and RAS-MAPK pathways, respectively. The consistent presence of IGF1R activity in NB cells prompted us to further investigate the contribution of IGF1R RTK to cell growth and proliferation of ALK-driven NB cells. 

### 3.2. Differential Sensitivity to IGF1R Inhibition in ALK-Driven NBs

**Figure 2:**
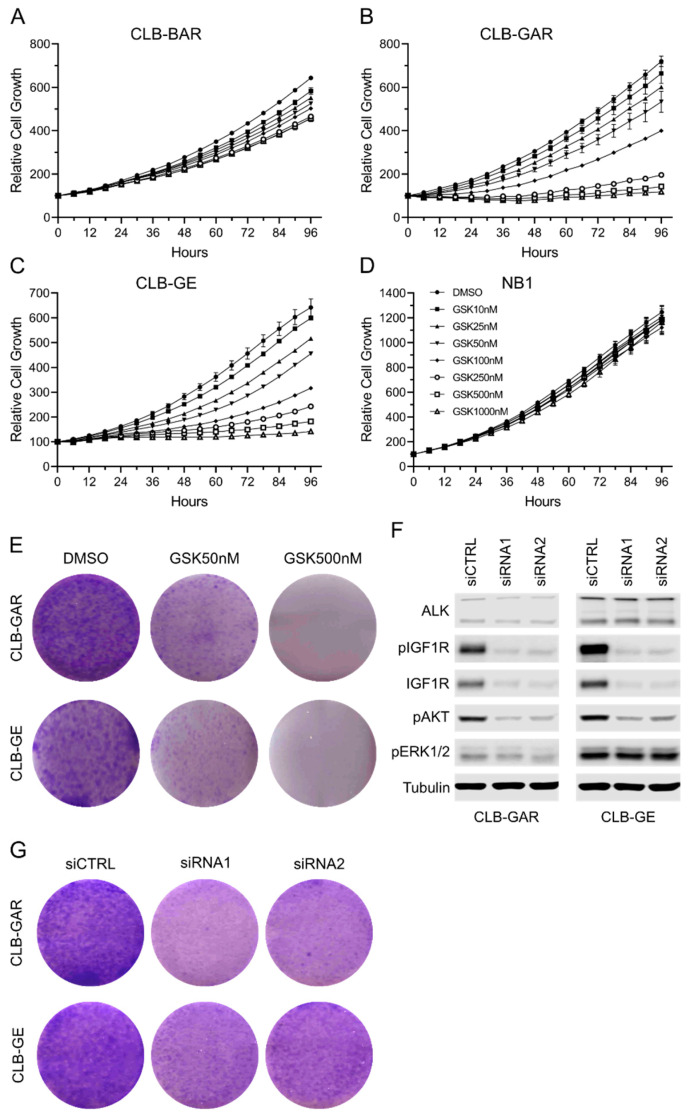
Sensitivity to IGF1R inhibition in ALK-driven NB cells. Cell growth of CLB-BAR (**A**), CLB-GAR (**B**), CLB-GE (**C**), and NB1 (**D**) in response to a dilution series of IGF1R inhibitor GSK1904529A over a time course of 96 h was monitored with Incucyte S3 Live-Cell Analysis System. Each curve represents one concentration of GSK1904529A (GSK for short) as indicated in (**D**). Colony formation assay of *ALK*-mutated NB cells after either inhibition or knockdown of IGF1R was also performed. (**E**) Colony formation assay showing the inhibitory effect on cell proliferation in CLB-GAR and CLB-GE treated with DMSO or GSK1904529A (50 nM and 500 nM). (**F**) Immunoblotting analysis of CLB-BAR and CLB-GE transfected with 50 nM of negative control siRNA (siCTRL), IGF1R siRNA1, or IGF1R siRNA2 and cultured for 48 h. The uncropped blots are shown in File S1. (**G**) Colony formation assay of CLB-BAR and CLB-GE transfected with negative control siRNA (siCTRL), IGF1R siRNA1, or IGF1R siRNA2 as indicated.

To test the contribution of IGF1R in ALK-driven NB cells, we treated CLB-BAR, CLB-GAR, CLB-GE, and NB1 cells with GSK1904529A, a selective and potent IGF1R inhibitor [52]. The SH-SY5Y cell line was not included in this assay because it contains a *KRAS* G12V mutation in addition to an *ALK* F1174L mutation [49]. Cell growth was monitored with Incucyte S3, and a differential sensitivity to IGF1R inhibition was observed among these four cell lines (Figure 2). CLB-BAR cells showed modest sensitivity and their growth was only slightly inhibited (Figure 2A). CLB-GAR and CLB-GE were sensitive to GSK1904529A treatment and exhibited dosage-dependent growth inhibition (Figure 2B,C). In contrast to these three cell lines, NB1 cells did not respond to IGF1R inhibition (Figure 2D). Similar sensitivity was also observed when another IGF1R inhibitor, linsitinib, was employed (Supplementary Figure S2). Interestingly, CLB-GAR and CLB-GE cells are *ALK*-mutated with relatively low ALK expression levels while CLB-BAR and NB1 cells are *ALK*-amplified, expressing high levels of the ALK RTK (Figure 1B), indicating that ALK expression status might determine the sensitivity to IGF1R inhibition in ALK-driven NB cells.

A colony formation assay was performed on CLB-GAR and CLB-GE cells treated with either 50 nM or 500 nM of GSK1904529A. After 14 days of culture, fewer and smaller colonies were observed in 50 nM GSK1904529A-treated groups compared with controls, and no obvious colonies were visible in 500 nM GSK1904529A-treated groups (Figure 2E). Knockdown of IGF1R with siRNAs was also performed on CLB-GAR and CLB-GE cells. Two independent and validated siRNAs effectively decreased IGF1R protein and phosphorylation levels without affecting ALK expression (Figure 2F). In both cell lines, IGF1R siRNA treatment led to decreased AKT activation. However, IGF1R knockdown showed only slight to modest effects on ERK1/2 phosphorylation (Figure 2F). Colony formation assays employing IGF1R siRNAs confirmed that depletion of IGF1R was able to reduce the growth of CLB-GAR and CLB-GE cells, as indicated with fewer and smaller colonies (Figure 2G). Taken together, IGF1R inhibition was able to inhibit the cell growth of NB cells harboring *ALK* mutations, such as CLB-GAR and CLB-GE.

### 3.3. IGF1R Inhibition Affects Downstream AKT and ERK1/2 Signaling

**Figure 3:**
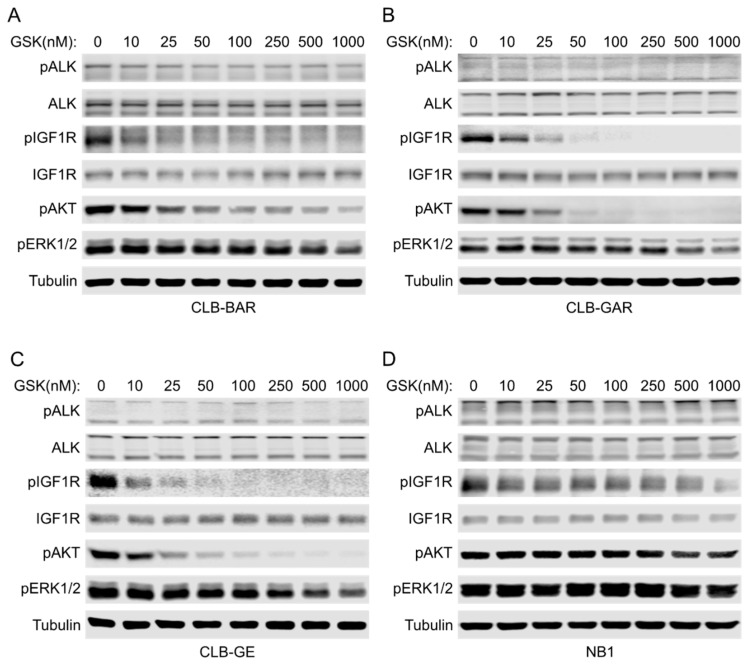
Effects of IGF1R inhibition on downstream AKT and ERK1/2 signaling. (**A**) ALK-driven CLB-BAR cells were treated with increasing concentrations of GSK1904529A (GSK for short) for 2 h and then harvested for immunoblotting analysis with antibodies as indicated. The same treatment and analysis were repeated with CLB-GAR (**B**), CLB-GE (**C**), and NB1 (**D**) cell lines. Phospho-ALK and phosphor-IGF1R antibodies were used to indicate ALK and IGF1R activation, respectively, and blots were stripped and reprobed to show the total protein levels. Phospho-AKT and phosphor-ERK1/2 antibodies were used to indicate the effects of IGF1R inhibition on downstream PI3K-AKT and RAS-MAPK signaling pathways. Tubulin was used as loading control. The uncropped blots are shown in File S1.

To better understand the difference in sensitivity between *ALK*-mutated and *ALK*-amplified NB cell lines, we treated cells with increasing concentrations of GSK1904529A for 2 h and immunoblotted for downstream signaling events. No obvious changes in ALK phosphorylation and protein levels were observed upon GSK1904529A treatment, indicating high selectivity of GSK1904529A for IGF1R over ALK, despite their kinase domain similarity [13,14]. GSK1904529A treatment effectively blocked phosphorylation of IGF1R in a dosage-dependent manner in CLB-BAR, CLB-GAR, and CLB-GE cells (Figure 3A–C). IGF1R activity was effectively inhibited with less than 100 nM GSK1904529A in both CLB-GAR and CLB-GE. Although 10 nM GSK1904529A was able to significantly decrease the phosphorylation of IGF1R, pIGF1R (Y1135/1136) signal was still detectable in the presence of 1 µM GSK1904529A in CLB-BAR cells (Figure 3A). Downstream AKT and ERK1/2 phosphorylation was also decreased in a dosage-dependent manner (Figure 3A–C). In all three cell lines, decreased ERK1/2 phosphorylation was only significant at higher GSK1904529A concentrations. In contrast, decreased AKT phosphorylation was significant at lower GSK1904529A concentrations and followed a similar pattern as that of IGF1R phosphorylation.

In contrast, no significant decrease of phosphorylation of either AKT or ERK1/2 was seen in NB1 cells until the concentration of GSK1904529A was increased to 1 μM (Figure 3D). This is consistent with the lack of growth inhibition by GSK1904529A in NB1 cells. The reason why NB1 cells are unresponsive to GSK1904529A is not clear, but one possibility may be that ALK is so highly overexpressed that the contribution to downstream signal transduction from IGF1R is very minor, which may also occur in *ALK*-amplified CLB-BAR cells. Unexpectedly, phosphorylation of IGF1R (Y1135/1136) was barely blocked even with 1 μM GSK1904529A in NB1 cells (Figure 3D), which may reflect transactivation as a result of excess ALK activity, as observed for the RET RTK in this cell line [44]. Taken together, ALK-driven NB cells show differential sensitivity to IGF1R inhibition, which correlates with ALK expression levels.

### 3.4. Combined Inhibition of ALK and IGF1R Synergistically Blocks Cell Proliferation and Promotes G1/S Phase Cell Cycle Arrest

ALK is a known oncogenic driver for ALK-driven NB cell lines including CLB-BAR, CLB-GAR, CLB-GE, and NB1, and inhibition of ALK activity with ALK inhibitors has been shown to block their proliferation [26,27,28,34]. Since we observed that IGF1R inhibition was also able to block cell proliferation, we hypothesized that IGF1R inhibition may enhance the anti-proliferation efficacy of ALK inhibitors synergistically in ALK-driven NB cells.

**Figure 4:**
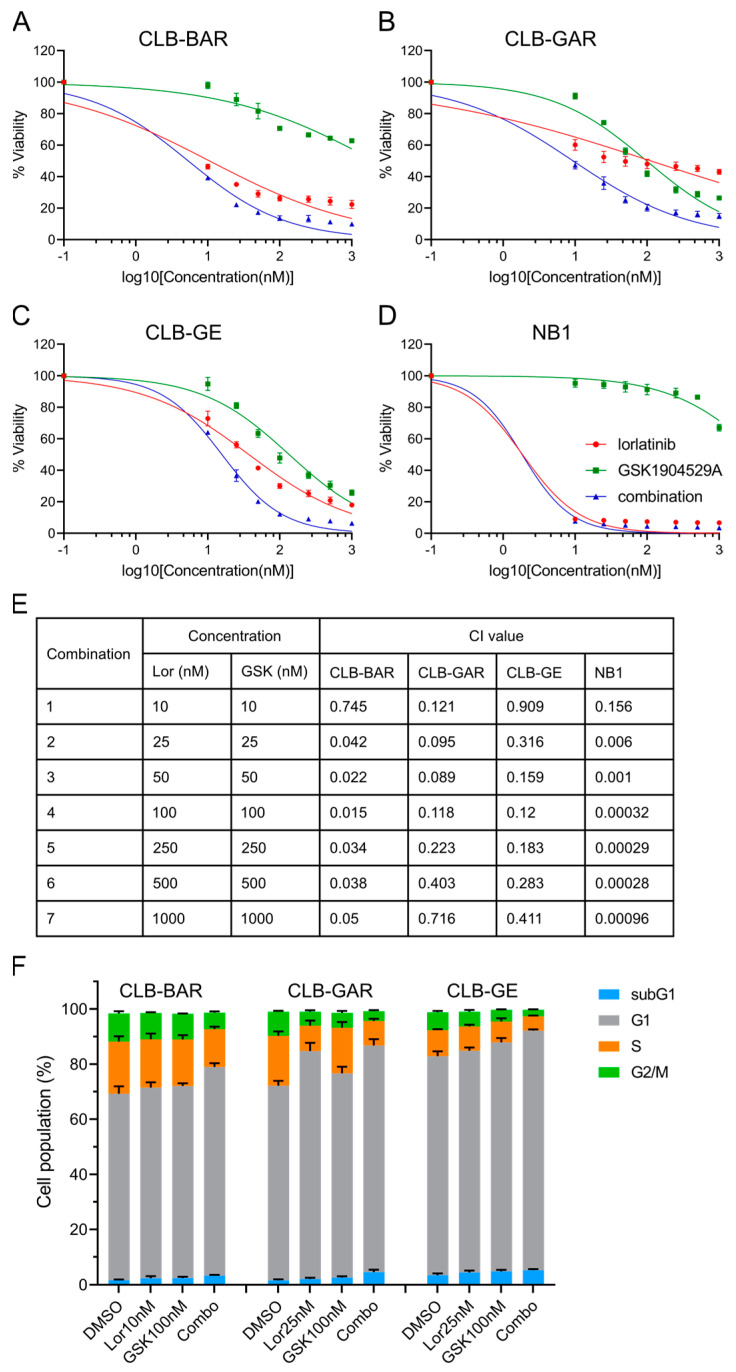
Synergy between lorlatinib and GSK1904529A, as well as treatment effects on cell cycle progression in ALK-driven NB cells. (**A**) Cell viability analysis (resazurin) in CLB-BAR after 72 h treatment as indicated. The same treatment and analysis were repeated in CLB-GAR (**B**), CLB-GE (**C**), and NB1 (**D**) cell lines. Cell viability was normalized to DMSO treated controls. IC50 values are listed in (**E**) as mean ± S.D. from at least three independent experiments. (**F**) Population distribution of cells in different phases in CLB-BAR, CLB-GAR, and CLB-GE after 48 h treatment with lorlatinib (Lor), GSK1904529A (GSK), or combination (Combo) of concentrations as indicated. Cells treated with DMSO were used as controls. Percentages are shown as mean ± S.D. from at least three independent experiments.

To test this hypothesis, we treated ALK-driven NB cells with increasing concentrations of the ALK inhibitor lorlatinib, the IGF1R inhibitor GSK1904529A, or a combination of both for three days. First, a differential sensitivity to lorlatinib was seen among the four ALK-driven NB cell lines (Figure 4A–D), with NB1 showing the highest sensitivity (IC50: 1.9 ± 0.1 nM) (Figure 4D) and CLB-GAR showing the lowest sensitivity (IC50: 118.6 ± 43.8 nM) (Figure 4B). Sensitivity to GSK1904529A was in agreement with our previous observations (Figure 2A–D). Synergistic effects were observed in all four cell lines at all concentration pairs, as indicated by the combination index (CI) values [50] (less than 1 denotes synergism) (Figure 4E), even though NB1 cells were highly sensitive to lorlatinib single-agent treatment and insensitive to GSK1904529A (Figure 4D). Combination treatment with lorlatinib and a second IGF1R inhibitor, linsitinib, also synergistically decreased proliferation of CLB-BAR, CLB-GAR, and CLB-GE cells (Supplementary Figure S2). Taken together, our results indicate that both ALK and IGF1R activity may contribute to proliferation of ALK-driven NB cells, and combined inhibition is able to block their proliferation synergistically.

We next investigated the effect of either single-agent or combination treatment on cell cycle progression, treating cells for 48 h prior to cell cycle analysis. Treatment of CLB-BAR cells with either 10 nM lorlatinib or 100 nM GSK1904529A, concentrations that effectively block phosphorylation of ALK or IGF1R, respectively, did not affect cell cycle progression significantly, whereas a significant increase in the G1 phase population (from 67.6% to 75.6%, *p* < 0.05, two-tailed paired student’s *t*-test) was seen in response to combination treatment (Figure 4F). In CLB-GAR cells, 25 nM lorlatinib alone resulted in a significant increase of the G1 phase population (from 70.6% to 82.7%, *p* < 0.001, two-tailed paired student’s *t*-test), while 100 nM GSK1904529A only slightly increased the G1 population (from 70.6% to 74%, *p* < 0.05, two-tailed paired student’s *t*-test). Combination of 100 nM GSK1904529A with 25 nM lorlatinib did not further increase the G1 population (82.2 ± 2.3%) when compared to lorlatinib treatment alone (82.7 ± 3.0%). However, combined lorlatinib/GSK1904529A treatment resulted in a significant increase in the subG1 population (from 1.6% to 4.6%, *p* < 0.05, two-tailed paired student’s *t*-test) when compared to lorlatinib (from 1.6% to 2.1%, *p* = 0.116, two-tailed paired student’s *t*-test) or GSK1904529A (from 1.6% to 2.7%, *p* < 0.05, two-tailed paired student’s *t*-test) single-agent treatment (Figure 4F). Combination treatment significantly increased the population of the G1 phase (from 79.5% to 86.9%, *p* < 0.05, two-tailed paired student’s *t*-test) in CLB-GE cells, while single-agent treatment at indicated concentrations did not effectively arrest cells at the G1/S phase, as seen in CLB-BAR cells (Figure 4F). In all three cell lines, combination treatment induced more apoptosis when compared to control or single-agent treatment (*p* < 0.05, two-tailed paired student’s *t*-test). Similar results were also obtained with lorlatinib and a second IGF1R inhibitor, linsitinib (Supplementary Figure S3A). To test whether IGF1R activity contributes to anti-apoptotic effects, we treated CLB-BAR, CLB-GAR, and CLB-GE cell lines with higher concentrations of ALK inhibitor lorlatinib (100 nM for CLB-BAR and 200 nM for CLB-GAR and CLB-GE), either alone or together with 200 ng/mL of IGF-1, for 24 h. Full-length (FL) and cleaved (CL) PARP were measured, and their ratio, an indicator of apoptosis, was calculated. The presence of IGF-1 resulted in a slight reduction of both the CL-PARP/FL-PARP and CL-PARP/Tubulin ratios in all three cell lines, supporting a role for IGF1R in contributing to anti-apoptotic activity in these NB cell lines (Supplementary Figure S3B).

### 3.5. Both ALK and IGF1R Contribute to Downstream AKT and ERK1/2 Signaling

To understand the underlying mechanisms of the observed ALK/IGF1R synergism, we treated CLB-BAR, CLB-GAR, and CLB-GE cells with lorlatinib, GSK1904529A, or both inhibitors in combination. We selected a concentration of each inhibitor able to effectively block phosphorylation of their target RTKs in the individual cell line (10 nM or 25 nM for lorlatinib, 50 nM for GSK1904529A), as well as a higher (10×) concentration (100 nM or 250 nM for lorlatinib, 500 nM for GSK1904529A). 

**Figure 5:**
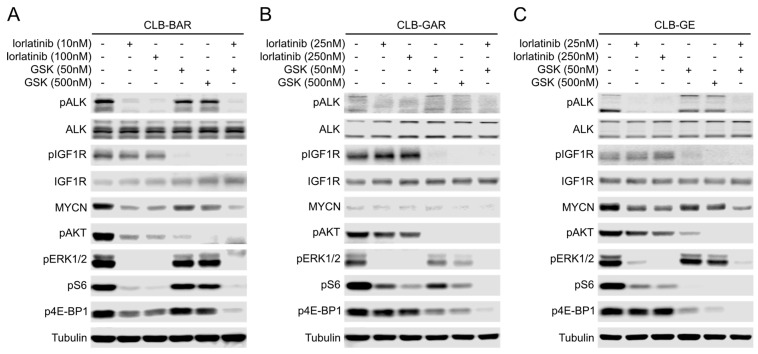
Activation of downstream AKT and ERK1/2 signaling by ALK and IGF1R. (**A**) Immunoblotting analysis of CLB-BAR cells treated with inhibitors as indicated for 2 h. ALK and IGF1R activity detected by pALK and pIGF1R antibodies, respectively, and total protein detected from the same blots after stripping. Phospho-AKT, phospho-S6, phospho-4E-BP1, and phospho-ERK1/2 were used to indicate the inhibitory effects on downstream PI3K-AKT-mTORC1 and RAS-MAPK signaling pathways. MYCN protein levels were also examined. Tubulin was used as loading control. The same treatment and analyses were repeated with CLB-GAR (**B**) and CLB-GE (**C**). The uncropped blots are shown in File S1.

In all three cell lines, when employed independently, lorlatinib and GSK1904529A (or linsitinib) at both concentrations only inhibited phosphorylation of the respective target RTKs, indicating their high selectivity (Figure 5; Supplementary Figure S4). In CLB-BAR, 10 nM lorlatinib effectively blocked ALK activation and abrogated phosphorylation of ERK1/2. Phosphorylation of AKT was largely inhibited at 10 nM lorlatinib but was still sustained at low levels even at 100 nM lorlatinib (Figure 5A). In contrast to lorlatinib, GSK1904529A treatment at both concentrations dramatically reduced the phosphorylation of AKT but was less effective in reducing phosphorylation of ERK1/2 even at 500 nM (Figure 5A). In CLB-GAR and CLB-GE, lorlatinib treatment effectively abrogated phosphorylation of ERK1/2 as observed in CLB-BAR, but was less effective in inhibition of AKT phosphorylation even at the higher concentration of 250 nM (Figure 5B,C). Treatment with 500 nM GSK1904529A abrogated phosphorylation of AKT in all three cell lines, but only blocked phosphorylation of ERK1/2 significantly in CLB-GAR (Figure 5B). In contrast, the combination of lower concentrations of lorlatinib and GSK1904529A was able to effectively abrogate both AKT and ERK1/2 phosphorylation, an effect that was not observed with single inhibitors even at 10× higher concentrations (Figure 5).

The mammalian target of rapamycin (mTOR), an atypical serine/threonine protein kinase, in the form of two protein complexes, mTORC1 and mTORC2, regulates multiple cellular processes. As a key component of the PI3K-AKT network, the mTORC1 complex regulates cell growth through its substrates 70-kDa ribosomal protein S6 kinase (p70S6K) and 4E binding protein 1 (4E-BP1) [53,54]. To examine how ALK and IGF1R activities affect AKT-mTOR signaling, phosphorylation of the 40S ribosomal protein S6 mediated by activated p70S6K, and phosphorylation of the eIF4E inhibitory protein 4E-BP1, were investigated. In CLB-BAR, lorlatinib treatment effectively abrogated phosphorylation of both S6 and 4E-BP1. In contrast, GSK1904529A treatment only resulted in modest decrease of phosphorylation of both proteins (Figure 5A). In CLB-GAR and CLB-GE, either lorlatinib treatment or GSK1904529A treatment effectively blocked the phosphorylation of S6, but only GSK1904529A treatment effectively blocked the phosphorylation of 4E-BP1 (Figure 5B,C). In all three cell lines, the combination of lower concentrations of lorlatinib and GSK1904529A was able to effectively abrogate both S6 and 4E-BP1 phosphorylation (Figure 5).

It is known that ALK regulates the initiation of transcription of *MYCN* through the PI3K-AKT-MEK5-ERK5 signaling axis [55,56], and the stability of MYCN through PI3K-AKT-GSK3β signaling axis [57,58,59] in ALK-driven/*MYCN*-amplified NB. CLB-BAR and CLB-GE are *MYCN*-amplified NB cell lines; therefore, we investigated the effect of lorlatinib and GSK1904529A on MYCN protein levels. Both lorlatinib and GSK1904529A single-agent short-term treatment resulted in decreased MYCN levels; however, GSK1904529A at both concentrations was less effective than lorlatinib. Combination lorlatinib/GSK1904529A treatment further reduced MYCN levels in CLB-BAR and CLB-GE cells (Figure 5A–C). We observed similar results using lorlatinib and a second IGF1R inhibitor, linsitinib (Supplementary Figure S4). Taken together, while both ALK and IGF1R contribute to activation of downstream signaling pathways, they show differential downstream activation of AKT and ERK1/2 signaling, and only combination treatment was able to abrogate both effectively.

### 3.6. Differential Preference for Downstream Adaptor Proteins

RTKs have multiple tyrosines in their intracellular domains that can be phosphorylated and serve as docking sites for Src-homology 2 domain (SH2)- and phosphorylated tyrosine binding (PTB)-domain containing adaptor proteins that determine the output of signal transduction [60]. The different inhibitory effects on downstream AKT and ERK1/2 signaling by ALK and IGF1R inhibitors prompted us to investigate the phosphorylation of adaptor proteins upon activation of ALK or IGF1R. CLB-BAR, CLB-GAR, and CLB-GE cells were stimulated with either ALKAL2 [61,62] or IGF-1 over a time course of 120 min to investigate the phosphorylation status of adaptor proteins involved in ALK and IGF1R signaling transduction including IRS2, GAB1, GAB2, and FRS2 [1,23].

**Figure 6:**
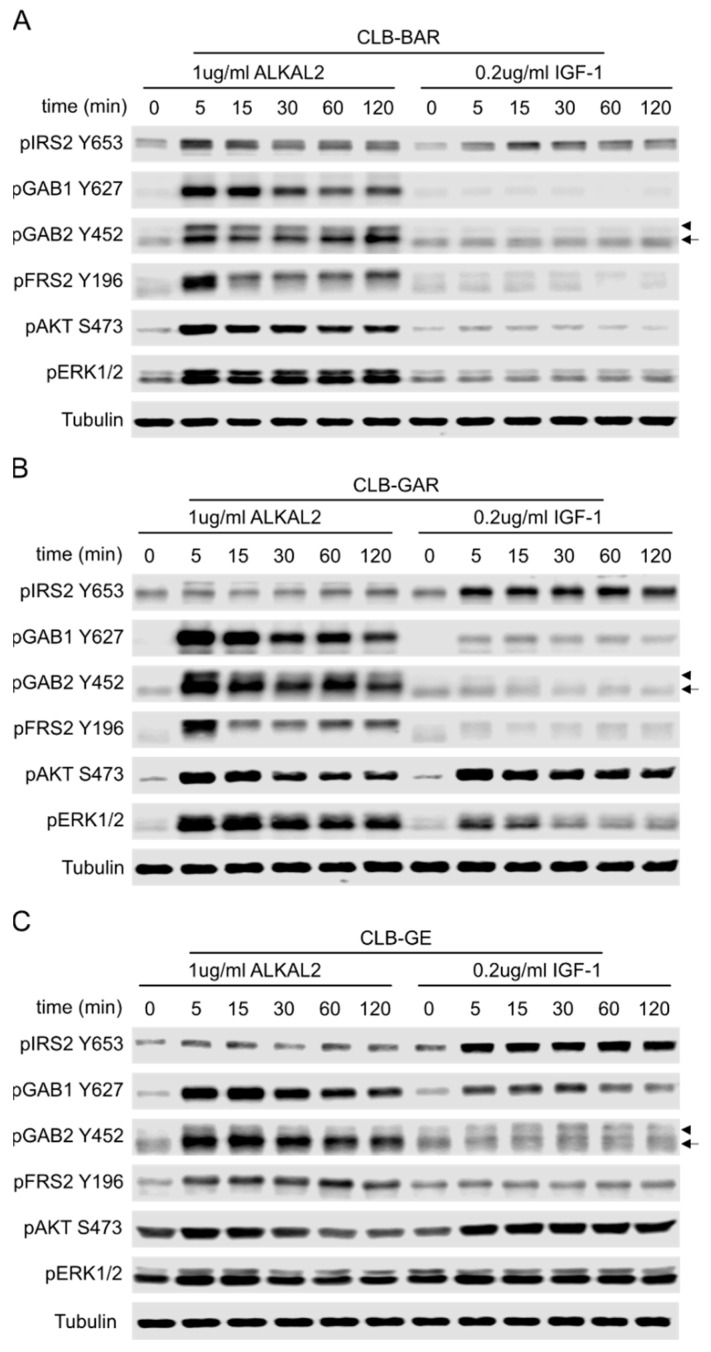
Selective phosphorylation of adaptor proteins by ALK and IGF1R. Immunoblotting analysis of CLB-BAR (**A**), CLB-GAR (**B**), and CLB-GE (**C**) stimulated with either 1 μg/ml ALKAL2 or 0.2 μg/ml IGF-1 for different times as indicated. Phosphorylation of adaptor proteins was investigated by immunoblotting for pIRS2 Y653 (p85 binding site), pGAB1 Y627 (SHP2 binding site), pGAB2 Y452 (p85 binding site), and pFRS2 Y196 (Grb2 binding site). Activation of downstream PI3K-AKT and RAS-MAPK signaling pathways was measured by pAKT S473 and pERK1/2. GAPDH was used as loading control. Arrow indicates phosphorylated GAB2 at tyrosine 452 and arrowhead indicates phosphorylated GAB1 due to cross reaction of the antibody to GAB1 when phosphorylated at tyrosines in the ‘YxxM’ p85 binding motif. The uncropped blots are shown in File S1.

In CLB-BAR, ALKAL2 stimulation of ALK led to increased phosphorylation of IRS2, GAB1, GAB2, and FRS2, whose signal intensity peaked at 5 min and then reduced gradually over time (Figure 6A). Downstream effectors, AKT and ERK1/2, exhibited similar phosphorylation dynamics as the adaptor proteins in response to ALK activation (Figure 6A). In contrast, IGF-1 stimulation resulted in robust phosphorylation of IRS2 but no detectable increase in phosphorylation of GAB1, GAB2, and FRS2. Accordingly, slightly enhanced activation of AKT but no obvious enhanced activation of ERK1/2 was observed (Figure 6A). 

In CLB-GAR (Figure 6B) and CLB-GE (Figure 6C) cells, ALKAL2 stimulation of ALK significantly enhanced the phosphorylation of GAB1, GAB2, and FRS2, with only modest effects on the phosphorylation of IRS2. In contrast, IGF-1 stimulation dramatically enhanced the phosphorylation of IRS2 and modestly enhanced the phosphorylation of GAB1 but not the phosphorylation of GAB2 and FRS2. Both ALKAL2 and IGF-1 were able to activate AKT strongly, but activation in response to IGF-1 was more sustained than that of ALKAL2 (Figure 6B,C). Given the contrast in phosphorylation patterns of IRS2 and comparable AKT activation after ligand stimulation, it is also reasonable to suggest that IRS2 is not the key adaptor protein for ALK, but is for the IGF1R in CLB-GAR and CLB-GE cells. ALKAL2 was able to induce strong activation of ERK1/2 in all three cell lines, whereas IGF-1 was only able to induce slight to moderate activation of ERK1/2 in CLB-GE and CLB-GAR, with no obvious activation of ERK1/2 in CLB-BAR (Figure 6). To further validate the role of IRS2 in the activation of the PI3K-AKT pathway upon stimulation with ALKAL2 or IGF-1, we performed immunoprecipitation experiments with either p85 or IRS2 antibodies. Unstimulated cells were used as control. IRS2 was able to recruit p85 on stimulation in all three cell lines; however, higher levels of p85 were observed upon stimulation with IGF-1 than with ALKAL2 in CLB-GAR and CLB-GE cell lines. In contrast, ALKAL2 stimulation resulted in increased p85 recruitment via IRS2 than IGF-1 stimulation in CLB-BAR cells (Supplementary Figure S5). This data is in agreement with the phosphorylation status of IRS2 upon either ALKAL2 or IGF-1 stimulation indicated by immunoblotting (Figure 6).

Taken together, these results show that ALK and IGF1R preferentially recruit and phosphorylate adaptor proteins, leading to differential activation of downstream AKT and ERK1/2 signaling in NB cells. The expression and phosphorylation levels of ALK may determine the importance of the IGF1R RTK in ALK-driven NB cells, where it might play an important role in driving cell growth and proliferation.

## 4. Discussion

In this study, we demonstrate a role for the IGF1R RTK in cell proliferation in *ALK*-mutated NB cells. Some NB cell lines harboring mutations in *ALK* are relatively insensitive to ALK inhibitors [26,27,28,31,49]. This may be because of the effect of *ALK*-activating mutations on inhibitor binding, resulting in reduced sensitivity to inhibitors [26,27,63]. An additional complicating factor is the complex genetic background in NB cell lines, for example, the SH-SY5Y cell line that has an *ALK* F1174L mutation also harbors an activating *KRAS* G12C mutation [49]. Another less-explored reason is the dynamic re-routing of signaling via other RTKs. In this study, we show that the IGF1R RTK is often activated in *ALK*-mutated NB cells. Our observations may explain the lack of ALK TKI efficacy on mTORC1 activation that has previously been reported in *ALK*-mutated *MYCN*-amplified NB cells [54]. In these NB cells, IGF1R or other RTKs activating mTORC1 may contribute to the PI3K-AKT-mTORC1-RSP6 signaling axis. 

**Figure 7:**
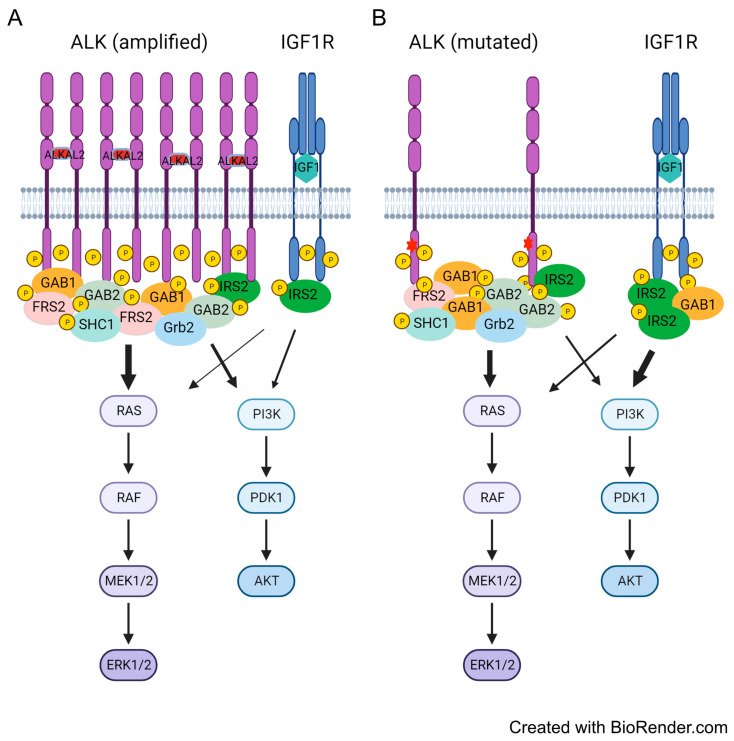
Schematic illustration of the differential contribution of ALK and IGF1R to downstream signaling in ALK-driven NB cells. (**A**) In *ALK*-amplified NB cells, excess ALK recruits and phosphorylates the majority of the adaptor protein pool as demonstrated. In contrast, the IGF1R RTK recruits and phosphorylates IRS2 protein. (**B**) In the *ALK*-mutated NB cells investigated here, ALK protein levels are lower than in *ALK*-amplified cells, and both ALK and IGF1R recruit and phosphorylate adaptor proteins. Due to selective recruitment of adaptor proteins and differences in expression levels, ALK and IGF1R contribute differentially to activation of downstream RAS-MAPK and PI3K-AKT pathways. Arrow thickness indicates contribution to downstream signaling pathways. ALK RTK in violet; IGF1R RTK in blue; red asterisks indicate activating mutations. Illustration was created with BioRender.com.

Adaptor proteins comprise vital components of the signaling machinery downstream of RTKs, with SH2-domain-containing and PTB-domain-containing adaptors playing critical roles connecting extracellular signals to intracellular biological effects [60]. Known adaptor proteins involved in ALK and IGF1R signaling transduction include IRS1, IRS2, GAB1, GAB2, CRK, FRS2, SHC1/2/3, etc. [22,23,48,64,65]. We show here that ALK preferentially engages GAB1, GAB2, and FRS2 on stimulation with ALKAL2 ligand, while activation of IGF1R robustly and preferentially engages IRS2. Interestingly, work from Emdal et al. reported a central role for IRS2 in ALK signaling transduction to downstream AKT signaling in NB1 cells that express high levels of ALK [64]. Indeed, activation of ALK in NB1 cells with ALKAL2 ligand also resulted in tyrosine phosphorylation of IRS2, although this was not as dramatic as that observed for, e.g., CRK [66]. Taken together, previous reports, together with our findings presented here, suggest that NB cells expressing lower levels of ALK employ IGF1R as their main IRS2-engaging RTK. In this case, both ALK and IGF1R contribute to the activation of downstream RAS-MAPK and PI3K-AKT signaling pathways. However, in NB cells expressing high levels of ALK, such as in *ALK*-amplified cell lines like NB1, ALK dominates, recruiting the majority of the adaptor proteins and activating signaling pathways (schematically illustrated in Figure 7). In agreement with this hypothesis, we observed in this study that NB1 cells are rather insensitive to both IGF1R inhibitors (GSK1904529A and linsitinib) employed in this study, with little effect on downstream AKT and ERK1/2 phosphorylation, suggesting that, in this NB cell line, the high expression levels of ALK may outcompete IGF1R in engagement of IRS2. In addition to IRS2, ALK also recruits GAB1, GAB2, SHC1, FRS2, and other adaptor proteins that link ALK activity to both PI3K-AKT and RAS-MAPK pathways, but preferentially the RAS-MAPK pathway (Figure 7).

Due to the high similarity in the kinase domain, inhibitors targeting IGF1R also target InsR. In this study, we employed two different IGF1R inhibitors, GSK1904529A and linsitinib, both of which target IGF1R and InsR with a similar range of IC50 values. Given the observation that both IGF1R and InsR activities are present in ALK-driven NB cells with a human phospho-RTK array, we cannot exclude the possibility that inhibition of InsR also leads to a decrease in cell proliferation. However, the signal intensity of InsR is weaker than that of IGF1R in these two ALK-driven NB cells. Moreover, it is believed that IGF1R signaling differs from InsR signaling even though both RTKs share common signaling pathways [1]. In addition, treatment with a specific humanized IGF-1R neutralizing antibody leads to a decrease in cell proliferation of NB cells like SH-SY5Y [67]. Most importantly, we employed siRNAs to knock down IGF1R, observing similar inhibitory effects on cell proliferation as observed with IGF1R inhibitors. Thus, we believe that the inhibitory effect on cell proliferation is most likely the result of inhibition of IGF1R upon treatment with IGF1R inhibitors.

In recent years, a number of preclinical studies have identified ALK combinatorial partners with potential for clinical efficacy [49,54,55,65,68,69,70]. Our data would suggest that IGF1R can also be added to this list. One concern regarding the use of IGF1R inhibitors in the clinic is whether or not they affect glucose uptake. A study with GSK1904529A shows that it potently reduces the phosphorylation of both IGF1R and InsR in vitro and in vivo, with no obvious effects on blood glucose levels at doses able to decrease tumor growth significantly [52]. Continuous administration of linsitinib in mouse xenograft models at certain doses elevates blood glucose levels and causes around 10% body weight loss, but these side effects disappear one week after the cessation of treatment [71]. Numerous clinical trials with linsitinib have demonstrated that linsitinib is well tolerated in patients [72,73,74,75,76]. Considering the synergistic effect observed in *ALK*-mutated NB cells in this study, further investigation of the effect of ALK/IGF1R inhibitor combination is warranted. 

## 5. Conclusions

Taken together, our results highlight the potential contribution of IGF1R to the proliferation of *ALK*-mutated NB cells. We further show that, mechanistically, both ALK and IGF1R drive the activation of downstream PI3K-AKT and RAS-MAPK pathways. However, while ALK contributes more to activating the RAS-MAPK pathway via adaptor proteins GAB1, GAB2, and FRS2, IGF1R shows biased activation of PI3K-AKT pathway via IRS2. Therefore, combined treatment with ALK and IGF1R inhibitors results in more effective anti-proliferation effects than single agents. In summary, our findings suggest that IGF1R plays a role in *ALK*-mutated NB, and that dual inhibition of these two RTKs may provide clinical benefit in treating this group of NB patients.

## Data Availability

The data presented in this study are available in this article and Supplementary Material.

## Supplementary Materials

The following supporting information can be downloaded at: https://www.mdpi.com/2072-6694/15/17/4252/s1. Figure S1. Layout of human phospho-RTK array and genetic background of NB cell lines. Figure S2. Dose response curve of lorlatinib and linsitinib as single agents or in combination in ALK-driven NB cells. Figure S3. Cell cycle analysis and effect of IGF1R activity on cell apoptosis. Figure S4. Inhibitory effects on downstream AKT and ERK1/2 signaling by lorlatinib and linsitinib as single agents or in combination. Figure S5. IRS2 recruits PI3K p85 subunit upon stimulation with ALKAL2 or IGF-1; File S1: Full pictures of the Western blots.
