# Supplementary material for: IGF1R Contributes to Cell Proliferation in ALK-Mutated Neuroblastoma with Preference for Activating the PI3K-AKT Signaling Pathway
Source: Cancers (Basel). 2023 Aug 25;15(17):4252. doi: 10.3390/cancers15174252 (PMC10563084; doi:10.3390/cancers15174252)
Supplement: Supplementary file 1 [file cancers-15-04252-s001.zip › Figures.pdf]

Supplementary Data

A

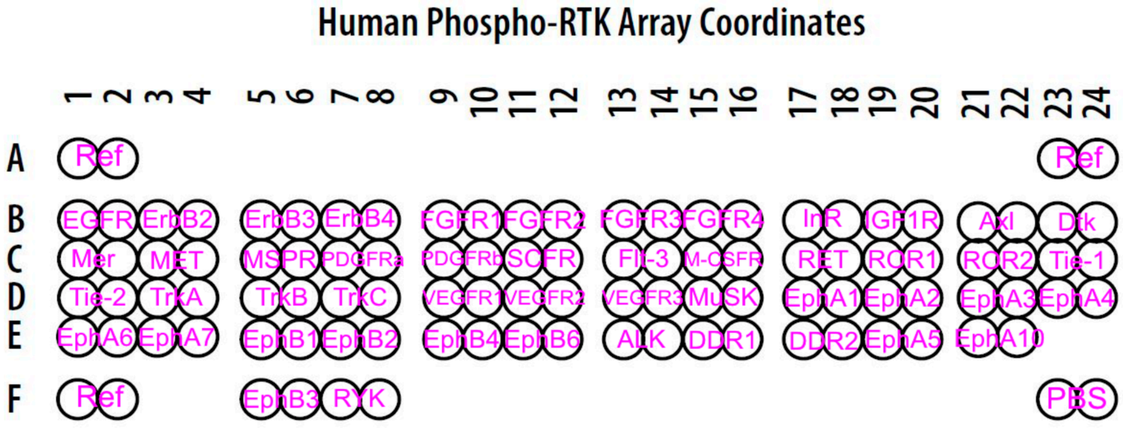

B

Cell lines	ALK	MYCN	TP53	RAS/MAPK
CLB-BAR	Amp, Δ ex4-11	Amp	WT	WT
CLB-GAR	R1275Q	Non-amp	WT	WT
CLB-GE	F1174V	Amp	WT	WT
NB1	Amp, Δ ex2-3	Amp	WT	WT
SH-SY5Y	F1174L	Non-amp	WT	KRAS G12V
SK-N-AS	WT	Non-amp	Mut	NRAS Q61K
BE2C	WT	Amp	Mut	NF1 low

Figure S1. Layout of human phospho-RTK array and genetic background of NB cell lines. (**A**) Layout of human phospho-RTK array adjusted from the manufacturer's manual. (**B**) Genetic features of *ALK, MYCN, TP53, NF1* and *RAS* in the NB cell lines used in this study. Amp: amplified; WT: wild type; mut: mutant.

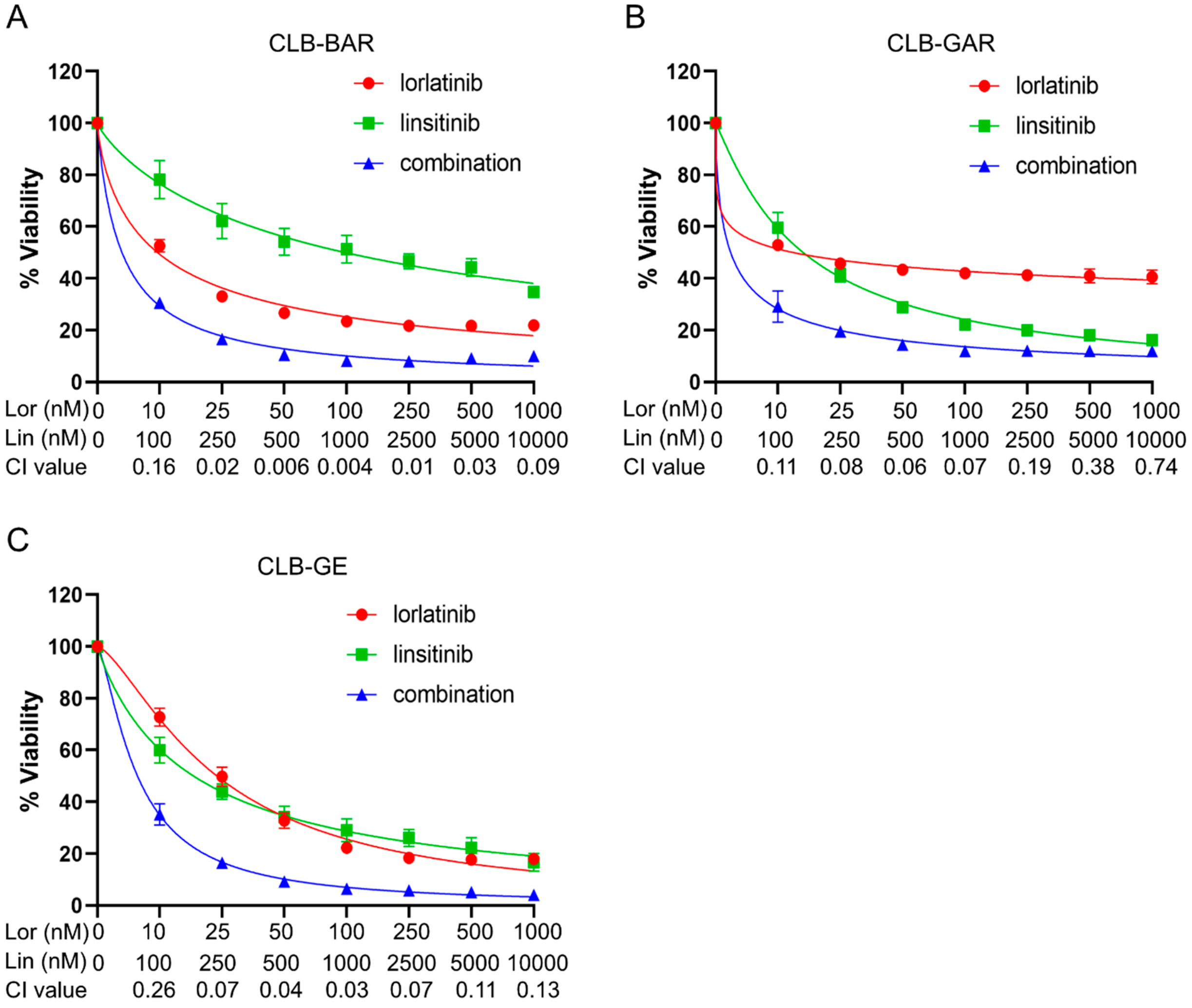

Figure S2. Dose response curve of lorlatinib and linsitinib as single agents or in combination in ALK-driven NB cells. (**A**) Cell viability analysis (resazurin) in CLB-BAR after 72 hours treatment as indicated (Lor: lorlatinib; Lin: linsitinib). The same treatment and analysis were repeated in CLB-GAR (**B**) and CLB-GE (**C**). Cell viability was normalized to DMSO treated controls. Synergistic effect of each combination was indicated by CI values listed under each chart. CI < 1 denotes synergism.

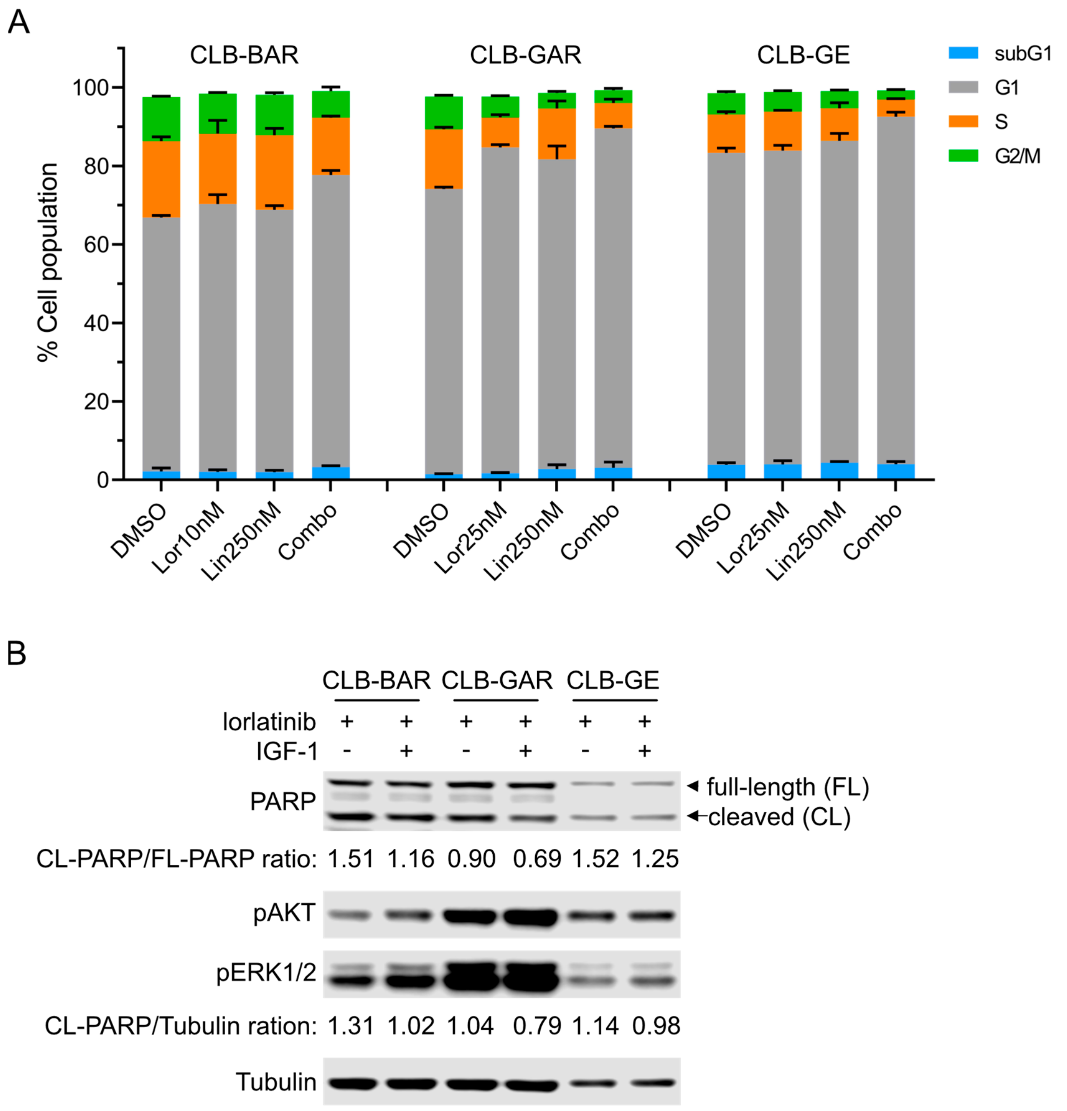

Figure S3. Cell cycle analysis and effect of IGF1R activity on cell apoptosis. (**A**) Population distribution of cells in different cell cycle phases in CLB-BAR, CLB-GAR and CLB-GE after 48 hours treatment with lorlatinib (Lor), linsitinib (Lin) or combination (Combo) of different concentrations as indicated. Cells treated with DMSO were used as blank controls. Percentages are shown as mean ± S.D. from at least three independent experiments. (**B**) CLB-BAR, CLB-GAR and CLB-GE cell lines were treated with higher concentrations of ALK inhibitor lorlatinib (100 nM for CLB-BAR and 200 nM for CLB-GAR and CLB-GE), either alone or together with 200 ng/ml of IGF-1, for 24 hours. Full-length (FL) and cleaved (CL) PARP were measured and their ratio, an indicator of apoptosis, was calculated. The presence of IGF-1 led to slight reduction in the CL-PARP/FL-PARP ratio in all three cell lines, indicating that IGF1R might contribute to anti-apoptotic activity in these NB cell lines. Calculation of CL-PARP/Tubulin ratios gave similar results. pAKT and pERK1/2 were used to measure downstream signaling.

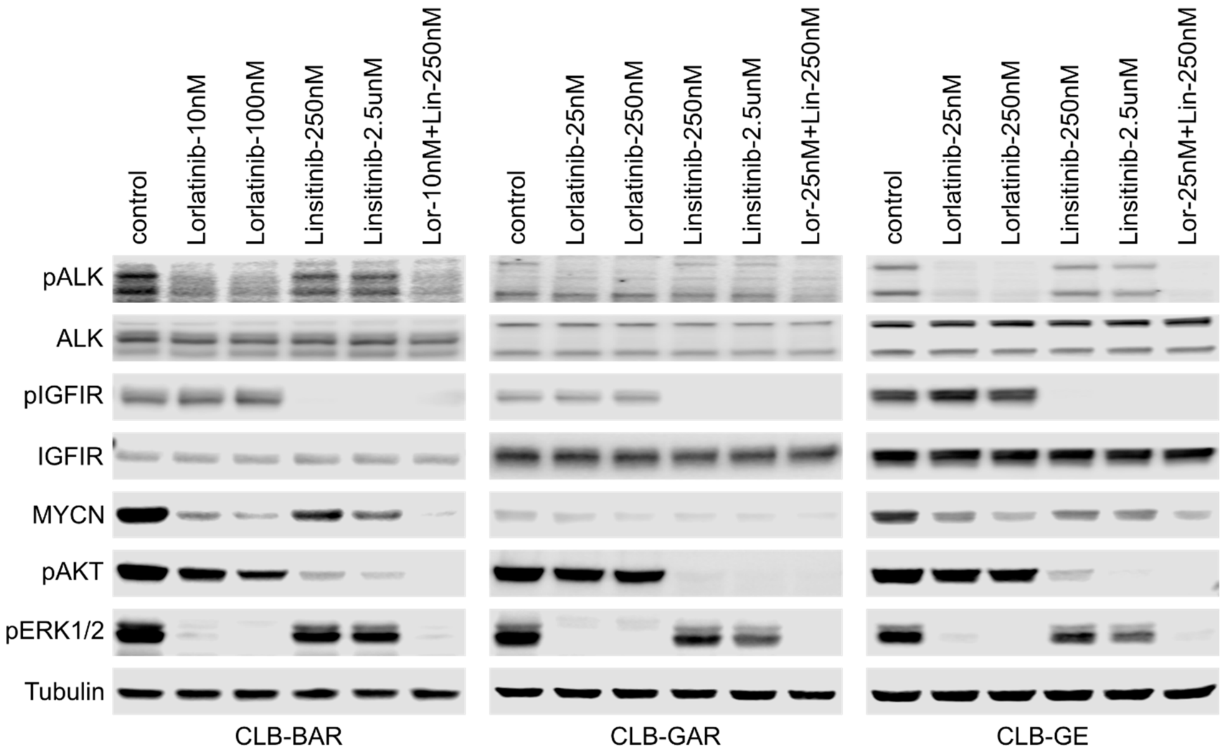

Figure S4. Inhibitory effects on downstream AKT and ERK1/2 signaling by lorlatinib and linsitinib as single agents or in combination. Immunoblotting analysis of CLB-BAR, CLB-GAR and CLB-GE treated with inhibitors as indicated for 2 hours. ALK and IGF1R activity was detected by pALK and pIGF1R antibodies respectively, and total proteins were detected from the same blots after stripping. Phospho-AKT and phosphor-ERK1/2 were used to indicate the inhibitory effects on downstream PI3K-AKT and RAS-MAPK signaling pathways. MYCN protein levels were also examined. Tubulin was used as loading control.

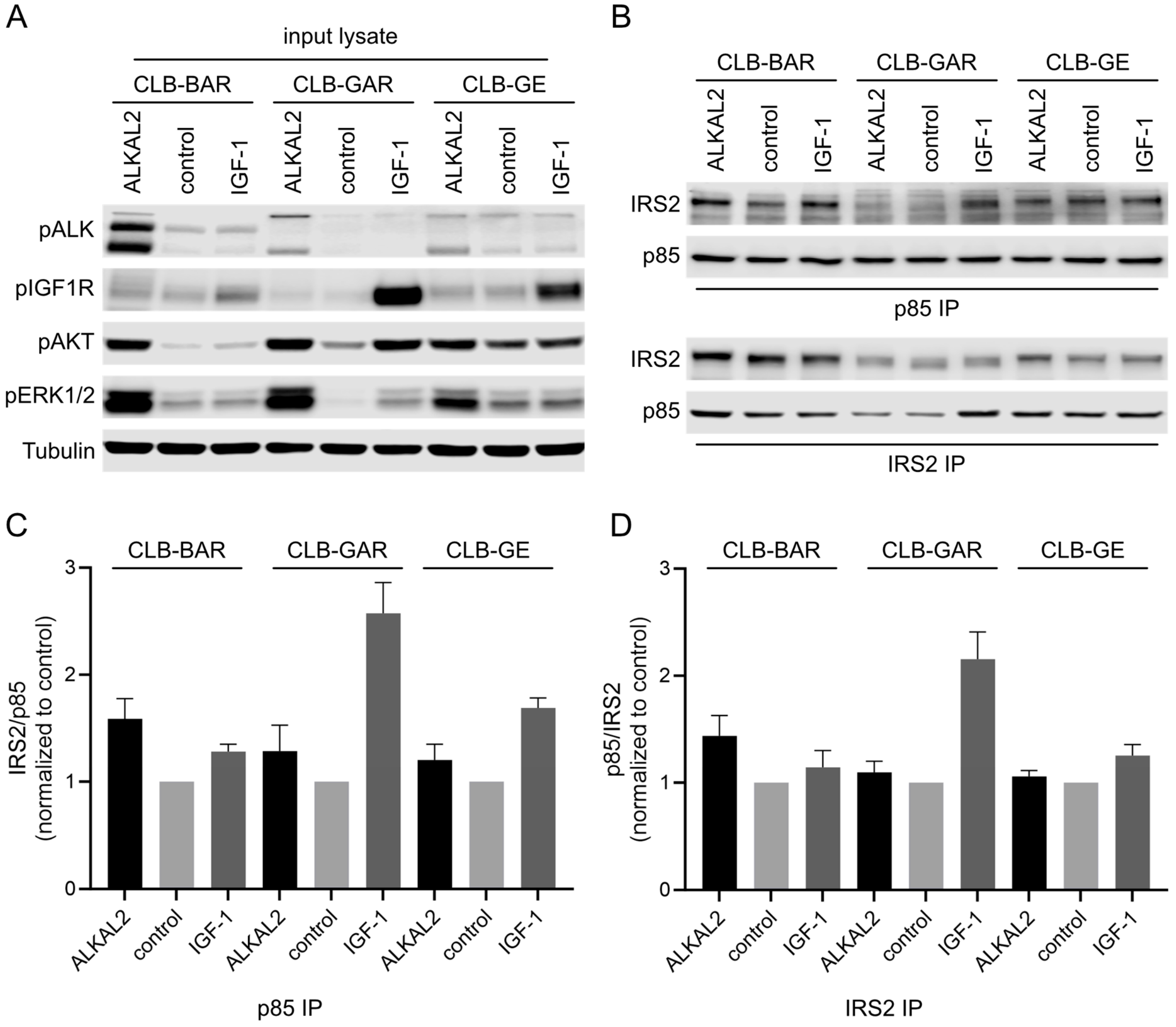

Figure S5. IRS2 recruits the PI3K p85 subunit upon stimulation with ALKAL2 or IGF-1. (**A**) Immunoblotting to validate the activation of ALK or IGF1R and downstream AKT and ERK1/2 signaling upon ALKAL2 or IGF-1 stimulation respectively. CLB-BAR, CLB-GAR and CLB-GE cell lines were stimulated with either 1 µg/ml ALKAL2 or 200 ng/ml IGF-1 for 15 minutes prior to lysis with RIPA buffer. Unstimulated cells were used as controls. Immunoprecipitation (IP) was performed with either anti-IRS2 antibody or anti-PI3 Kinase p85 antibody. IP products were subjected to immunoblotting analysis of IRS2 and p85 (**B**). The ratios of IRS2/p85 for p85 IP products (**C**) and p85/IRS2 for IRS2 IP products (**D**) were measured and normalized to control cells for each cell line. Data represents mean ± S.D. from at least three independent experiments.
