# Supplementary material for: IGF1R Contributes to Cell Proliferation in ALK-Mutated Neuroblastoma with Preference for Activating the PI3K-AKT Signaling Pathway
Source: Cancers (Basel). 2023 Aug 25;15(17):4252. doi: 10.3390/cancers15174252 (PMC10563084; doi:10.3390/cancers15174252)

# IGF1R-ALK-mutated NB

Raw data

Fig 1B. IGF1R acitivity is present in ALK-driven NB cells.

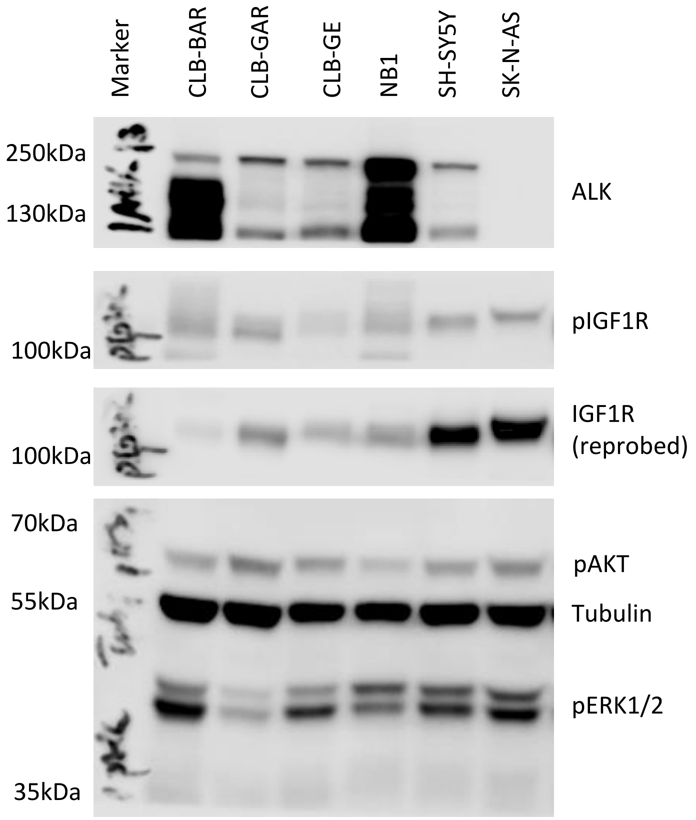


Revised figure

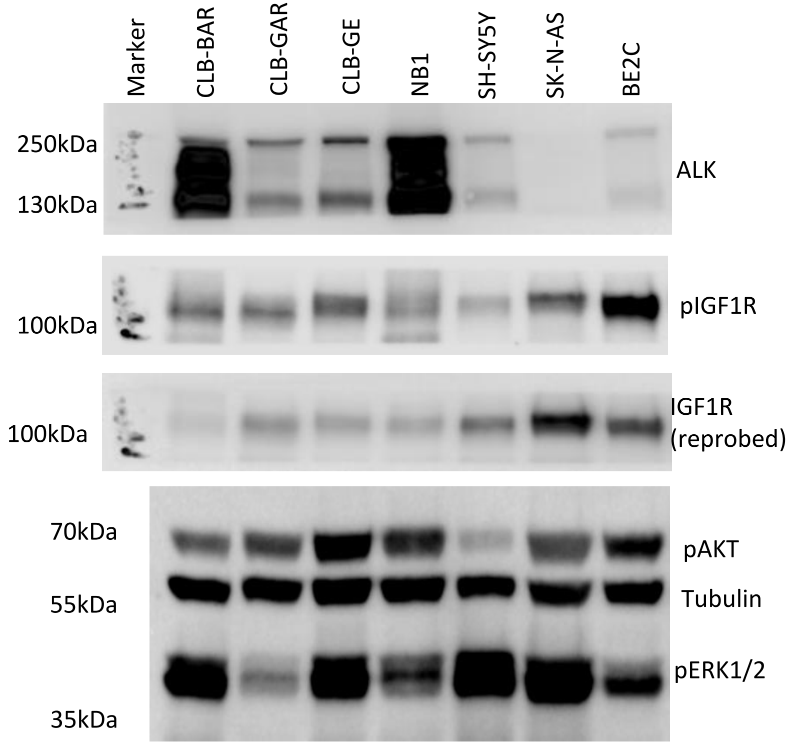

# Fig 2F. Effects of knockdown of IGF1R in CLB-GAR and CLB-GE

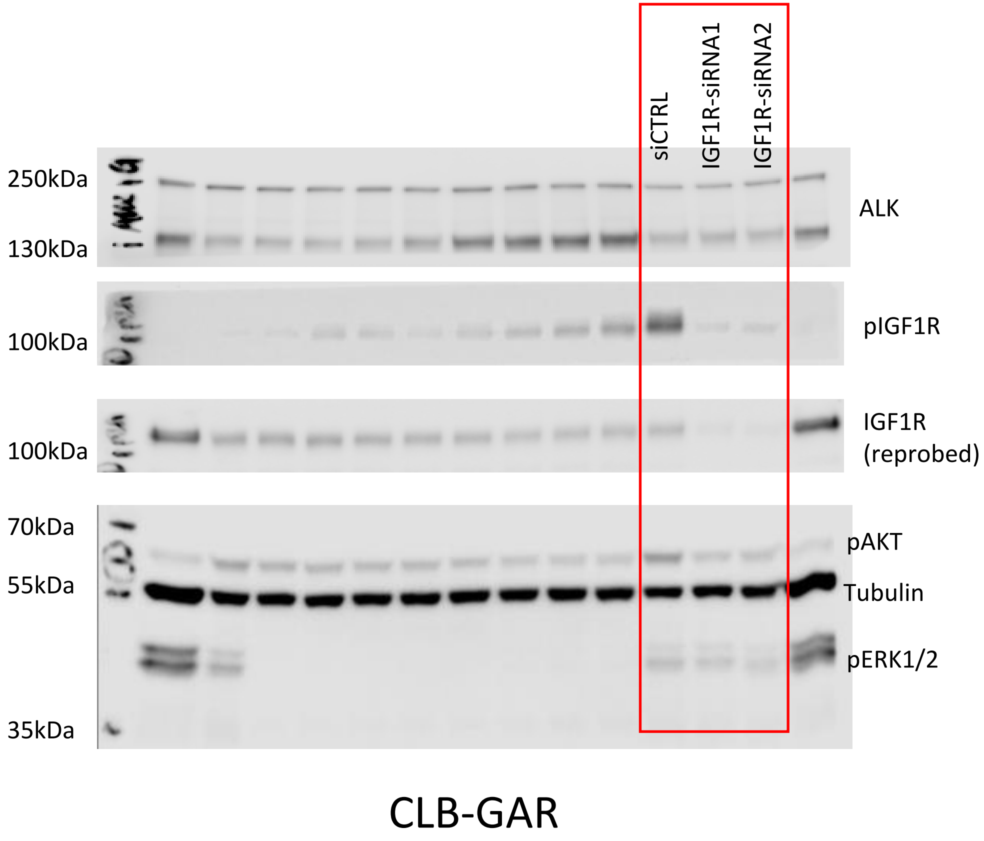


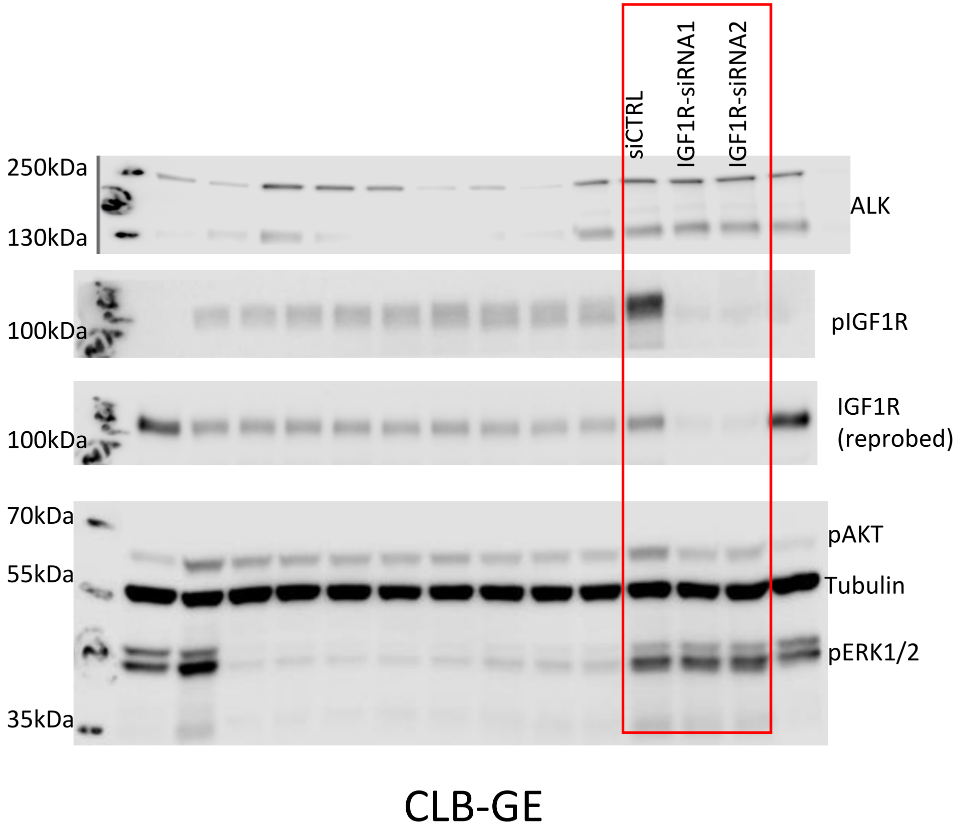

Fig 3A,B. BAR,GAR-GSK serial-inhibition

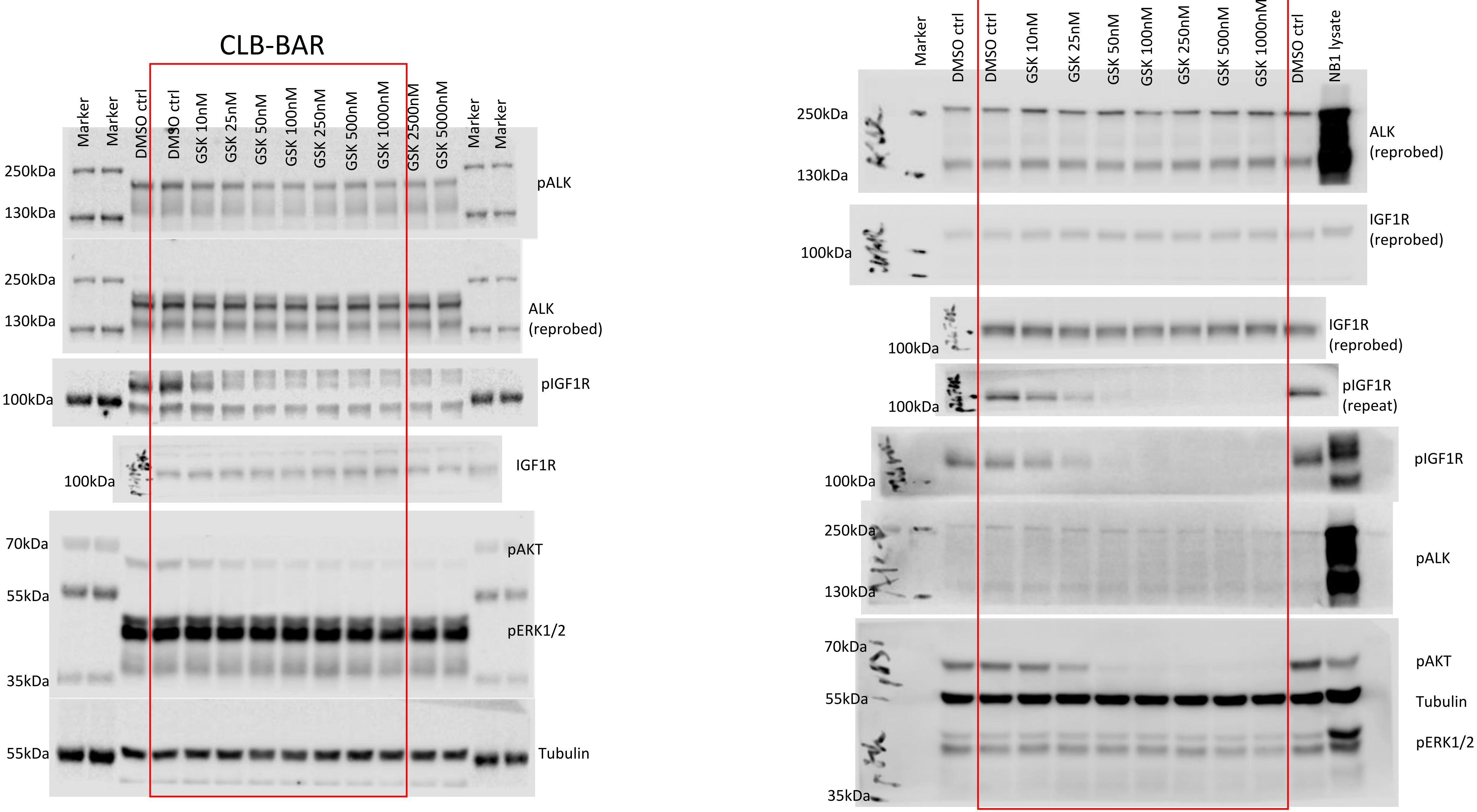

# Fig 3,D. CLB-GE, NB1-GSK serial-Inhibition

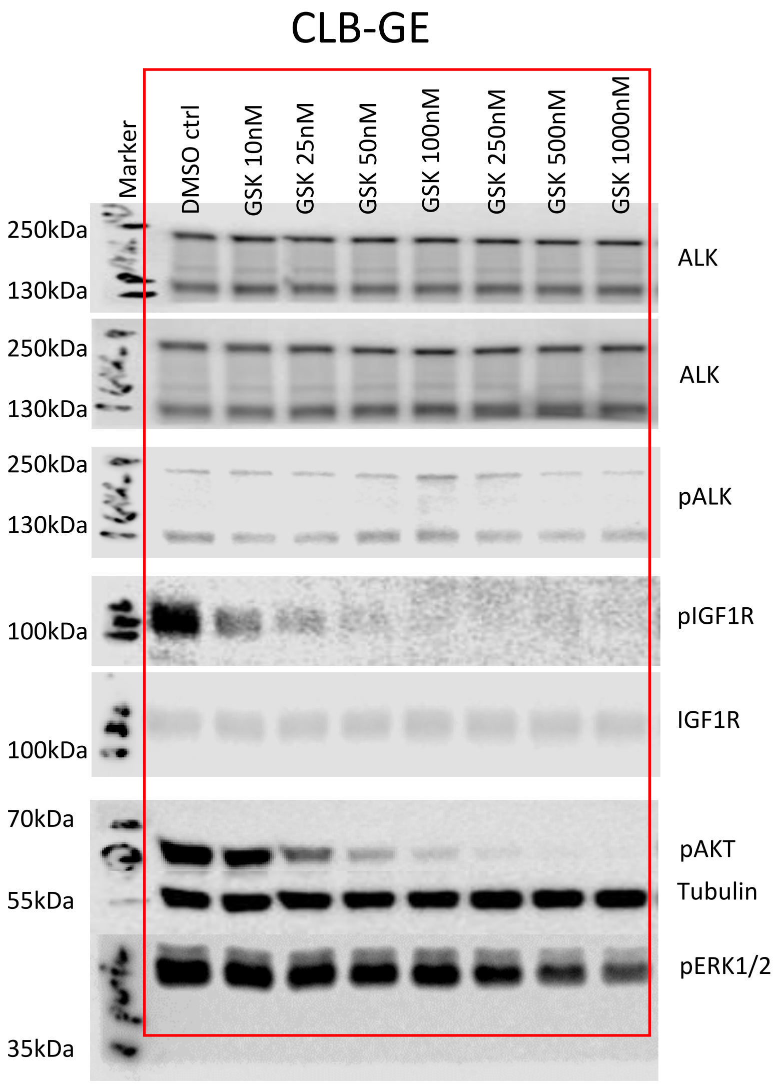


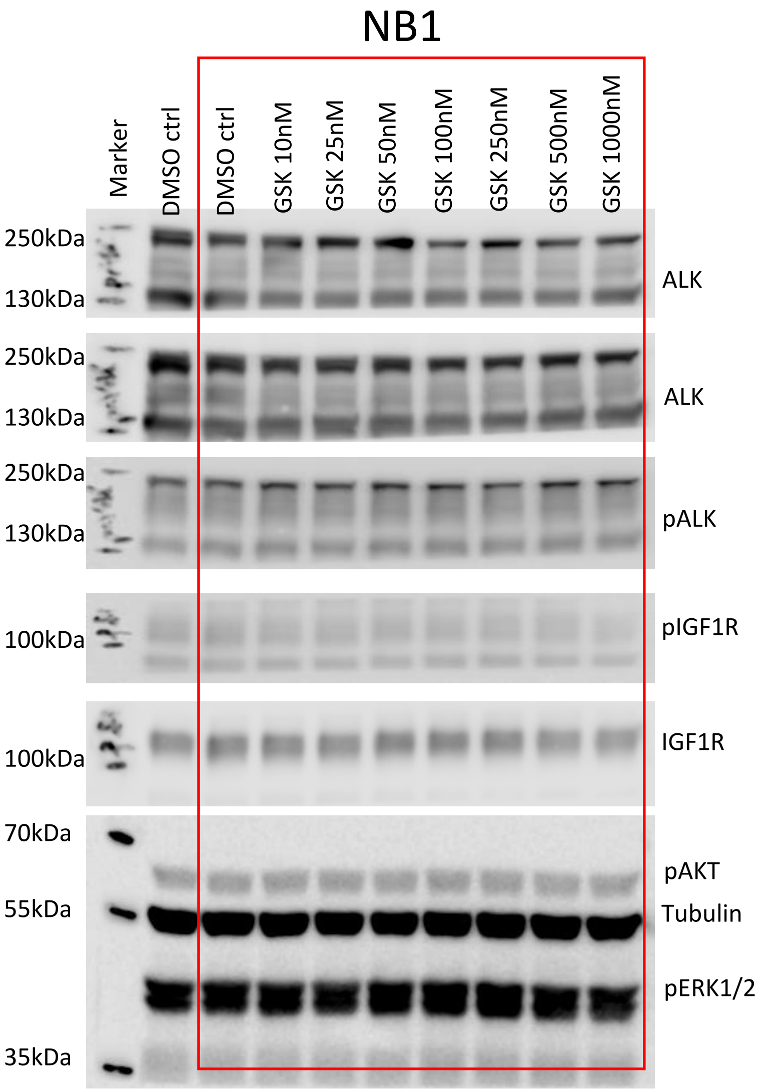

# Fig 5. Both ALK and IGF1R activities contribute to downstream signaling (2h)

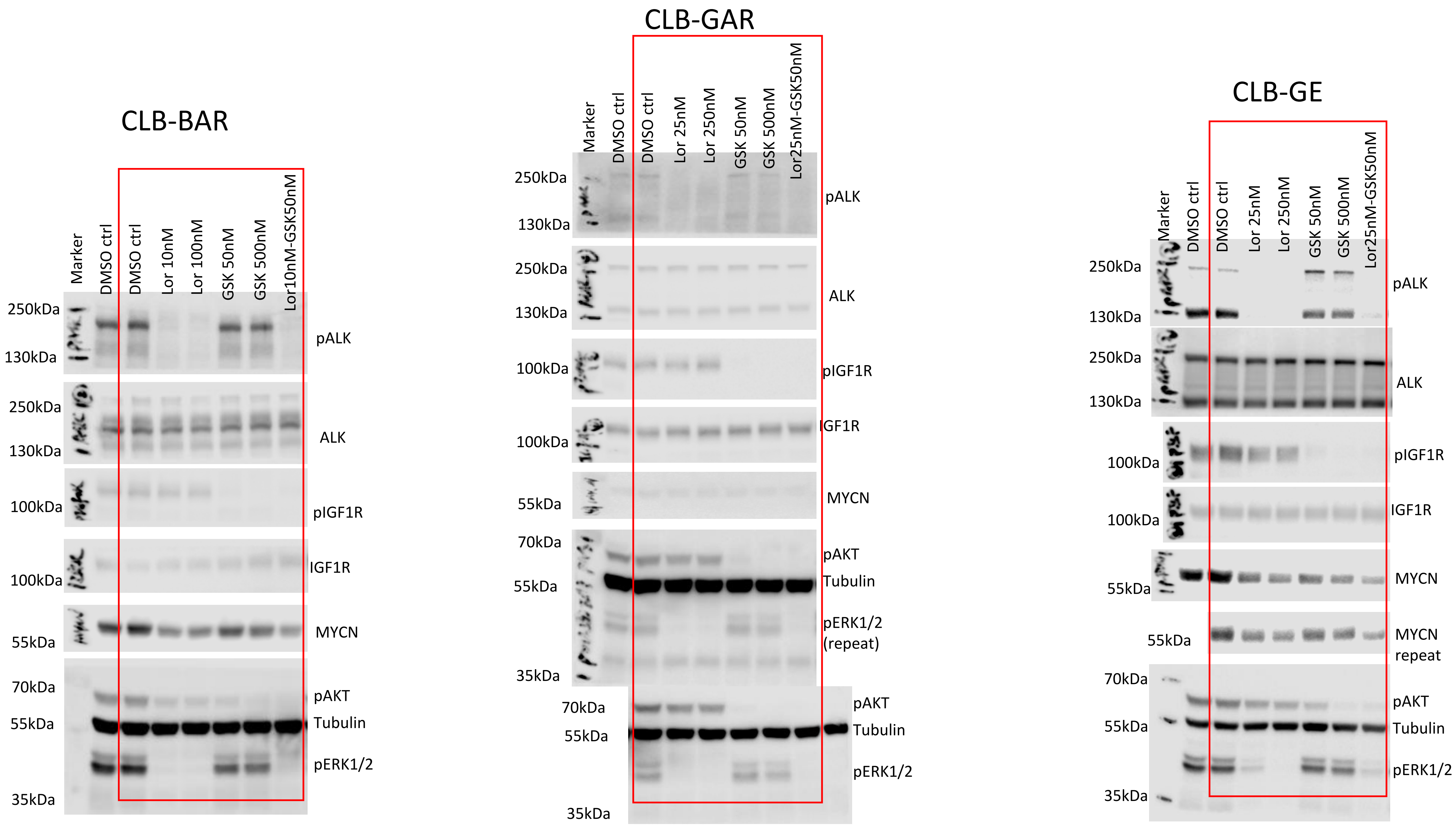

Fig 5. Both ALK and IGF1R activities contribute to downstream signaling (2h) (**for revision**)

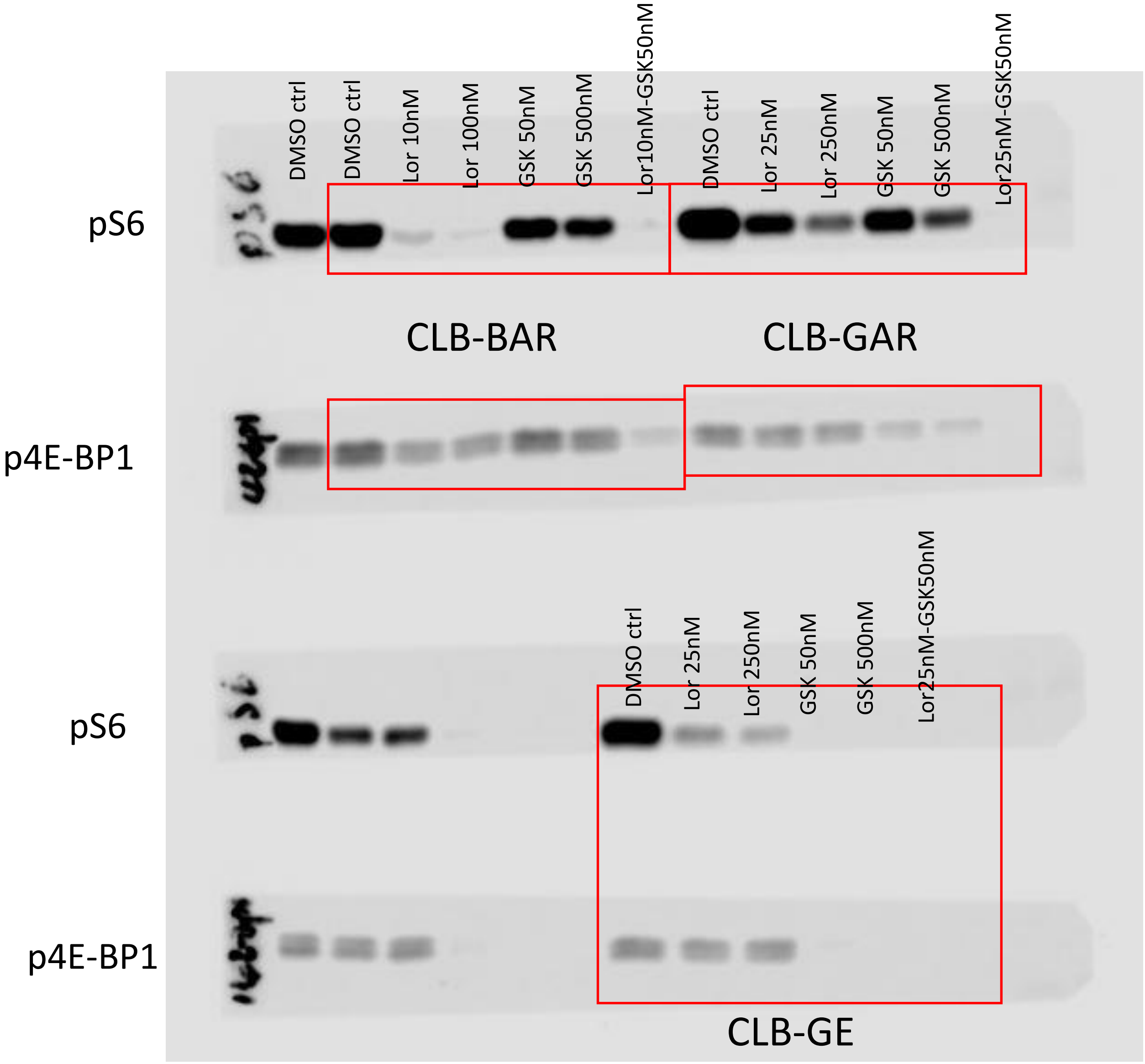

# Fig 6. Differential preference for downstream adaptors

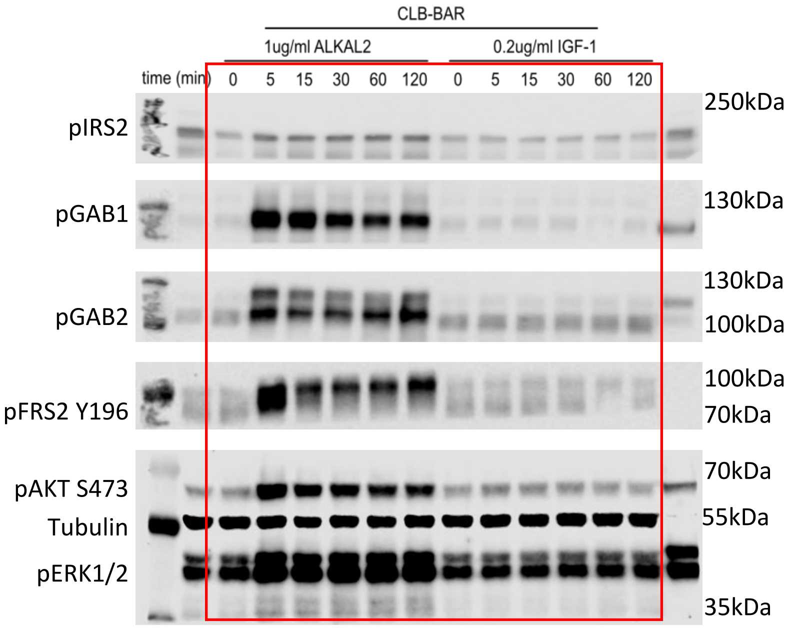


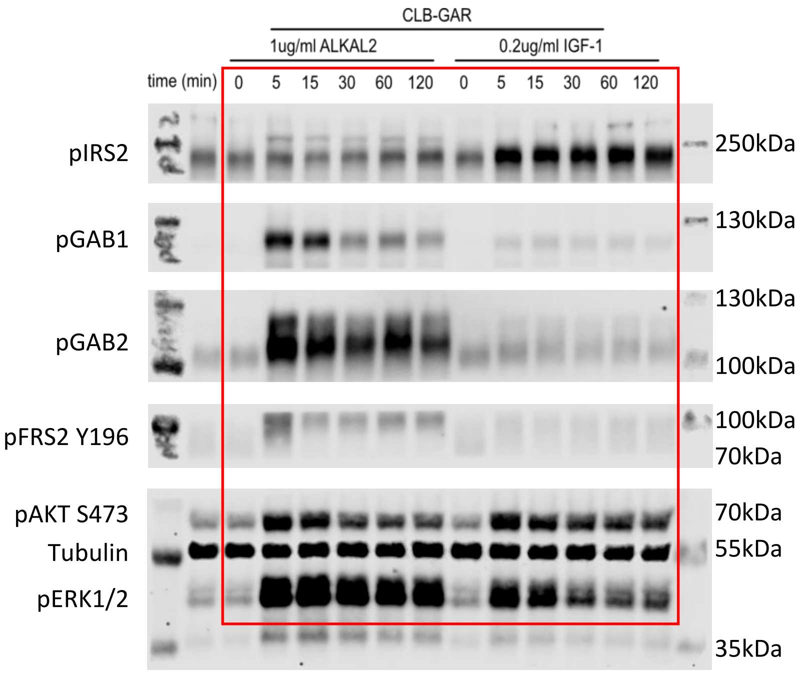


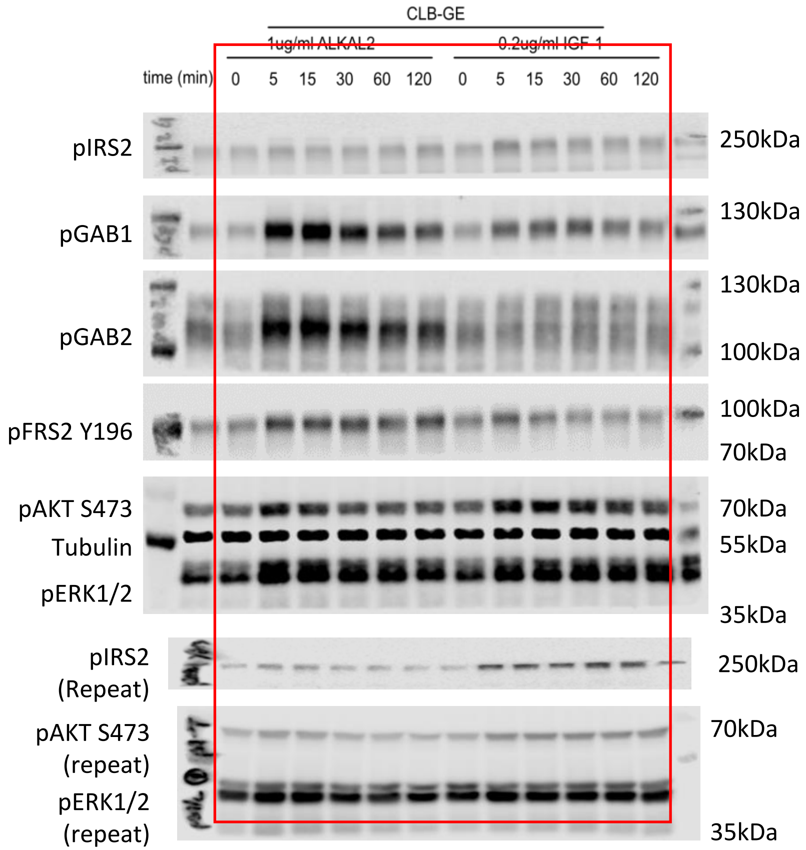

Fig S3B. Effect of IGF1R activity on cell apoptosis

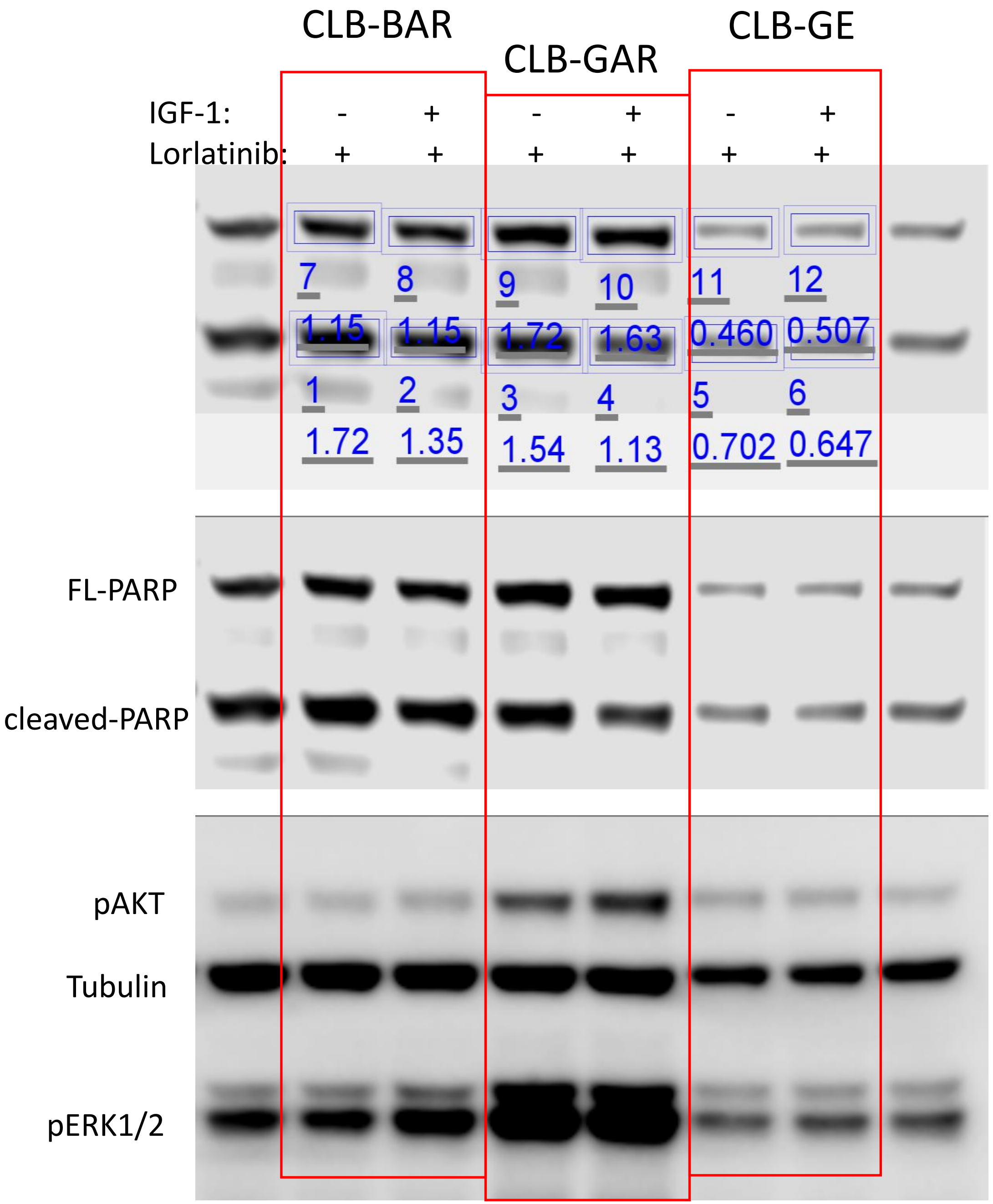

# Suppl. Fig4 Both ALK and IGFIR contribute to ALK-driven NBs

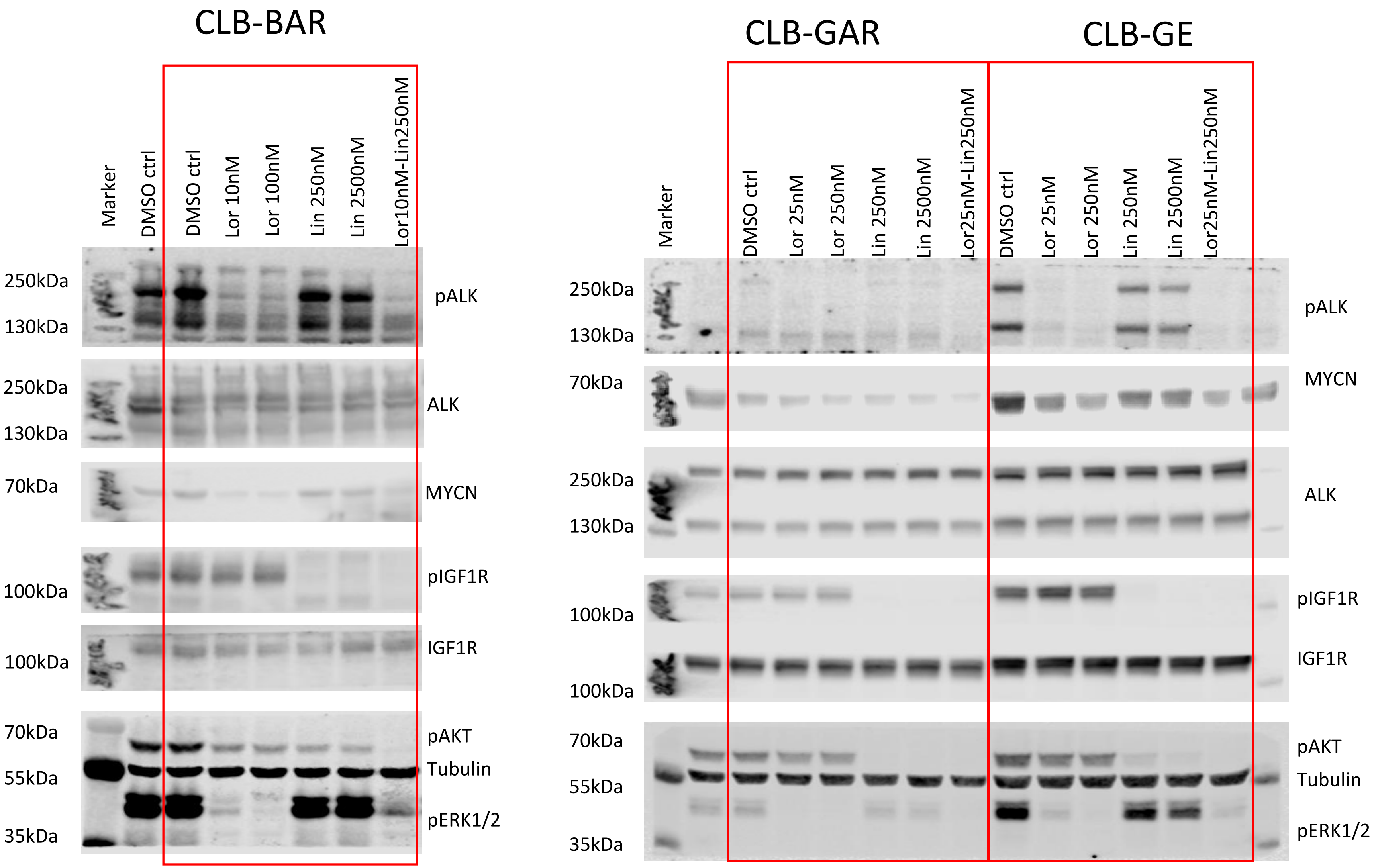

Figure S5. IRS2 recruits the PI3K p85 subunit upon stimulation with ALKAL2 or IGF-1.

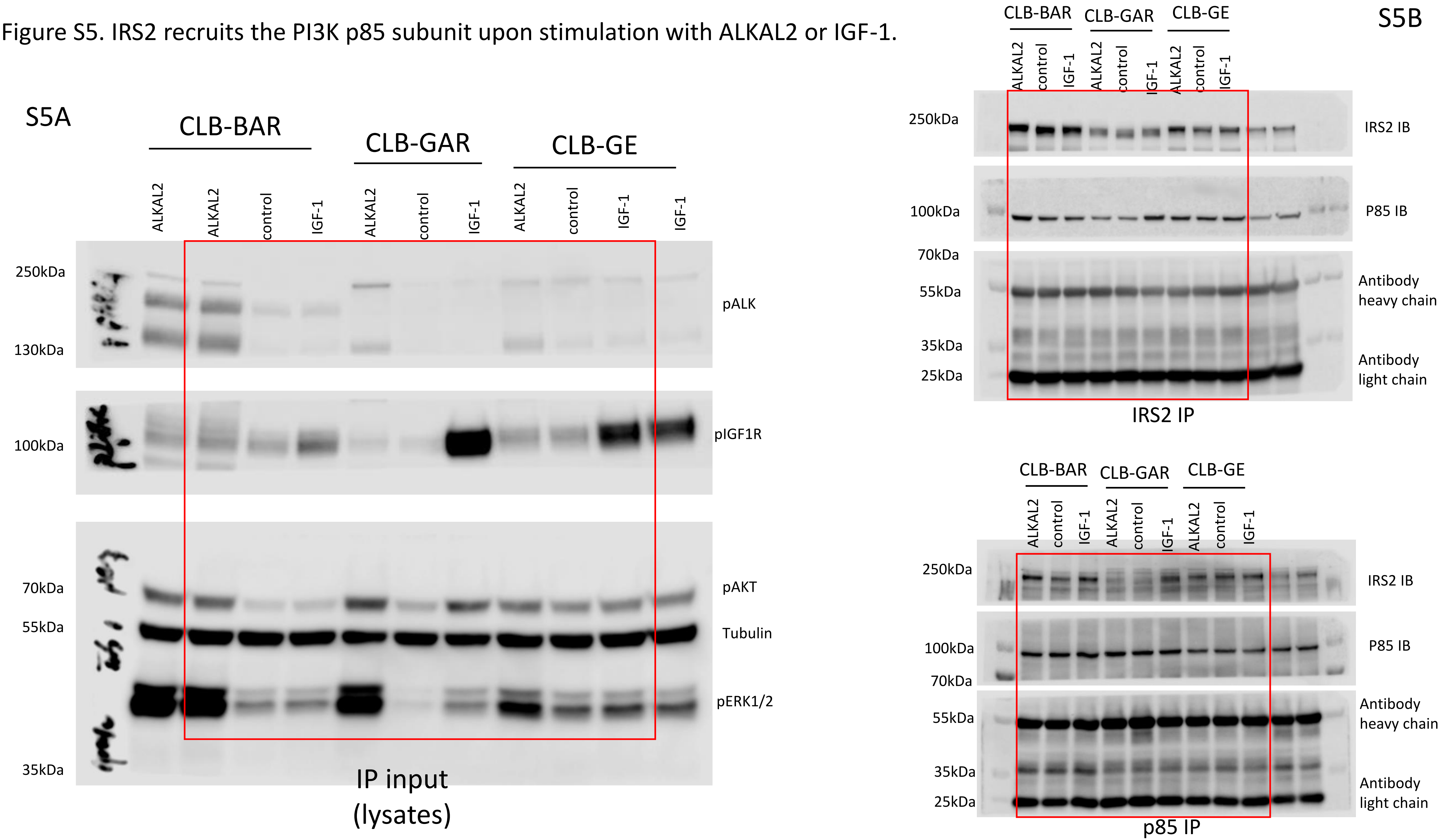

Supplement: Supplementary file 1 [file cancers-15-04252-s001.zip › File S1.pdf]
